# Supplementary material for: Combining discovery and targeted proteomics reveals a prognostic signature in oral cancer
Source: Nat Commun. 2018 Sep 5;9:3598. doi: 10.1038/s41467-018-05696-2 (PMC6125363; doi:10.1038/s41467-018-05696-2)
Supplement: Supplementary file 1 — Supplementary Information [file 41467_2018_5696_MOESM1_ESM.pdf]

Supplementary Information

Combining discovery and targeted proteomics reveals a prognostic signature in oral cancer

Carnielli et. al.

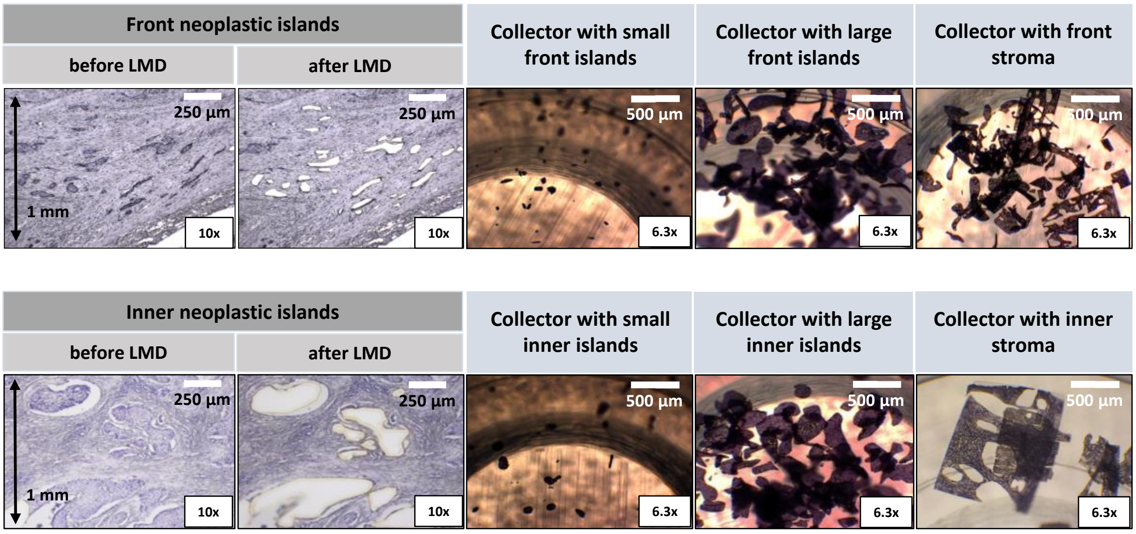

Supplementary Figure 1. Laser microdissection of neoplastic island and tumor stroma region from FFPE OSCC tissues. The ITF was delimited as 1 mm-depth from the edge of the tumor slice and the inner tumor was defined as up to 1 mm from the epithelial tumor tissue origin. The microdissected OSCC tissues were kept in the microtube lid.

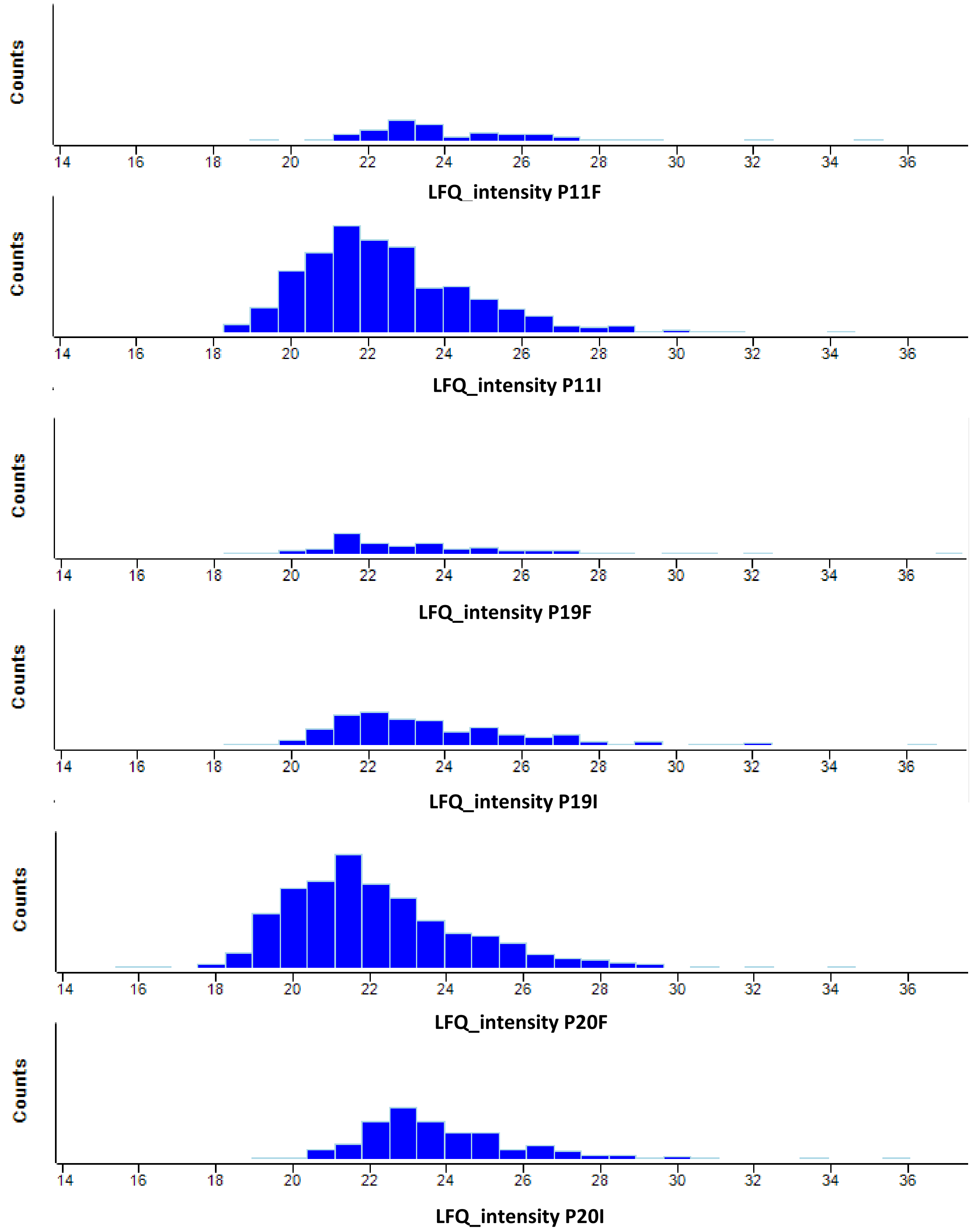

Supplementary Figure 2. Histogram of the samples of tumor stroma that were excluded from data analysis (P11, P19, P20). The overall low quantitation of proteins identified in these samples was used as exclusion criteria. Samples from the invasive tumor front are indicated by "F", and samples from the inner tumor are indicated by "I".

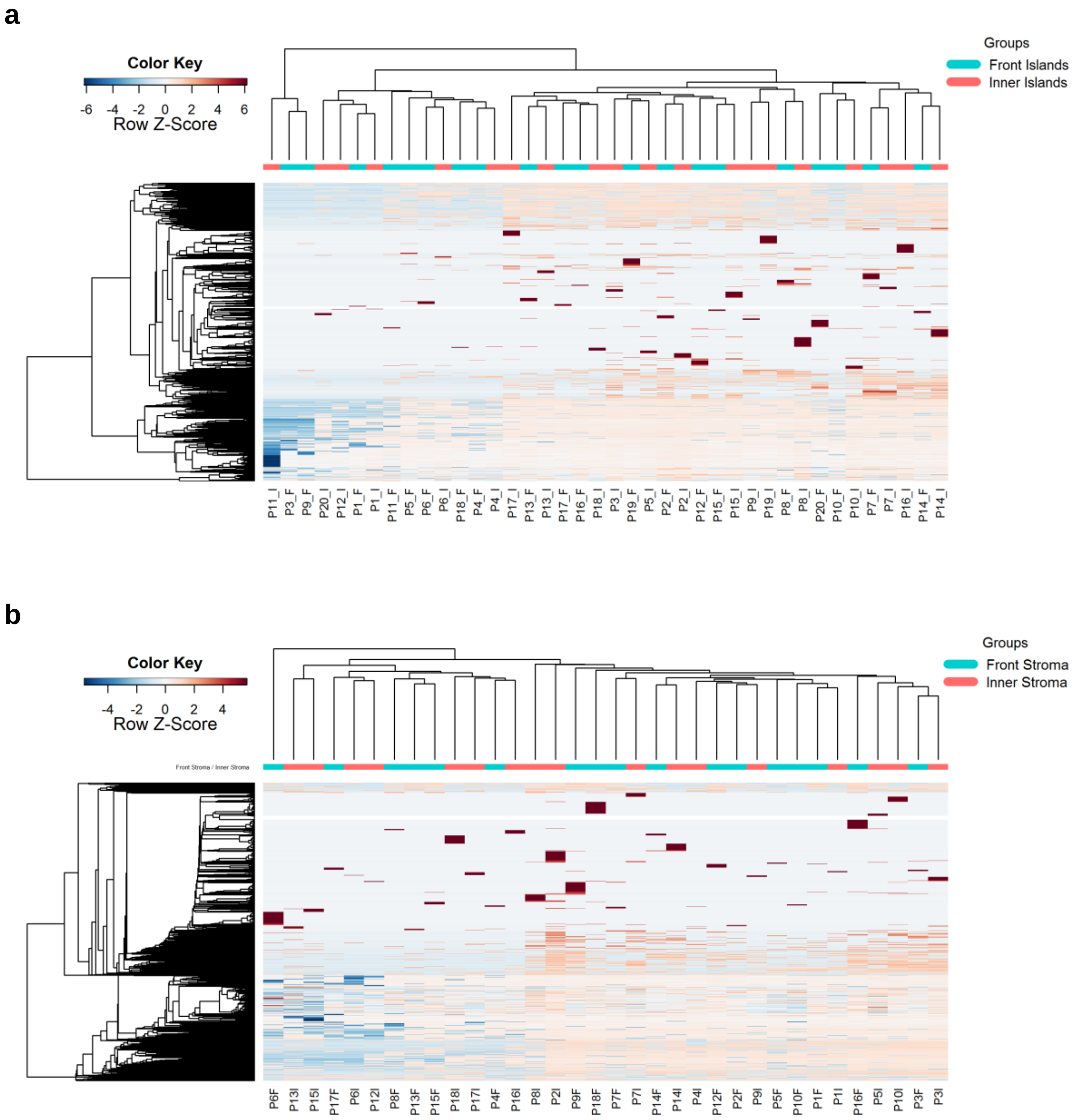

Supplementary Figure 3. Clustering analysis of all proteins identified in the invasive tumor front (ITF) and in the inner tumor of (a) neoplastic islands (20 patients) and (b) tumor stroma (17 patients). As observed, some proteins were only quantified in a few numbers of samples, as highlighted by the "gap" present in the middle area of the heat map. These entries were removed by the filter of valid values as shown in the figure 2 (main text). Values for each protein (rows) and for each microdissected sample (columns) are colored based on the protein abundance, in which high (red) and low (blue) values (Z-scored log2 LFQ intensity values) are indicated in the color scale bar at the top left. The colored bars above the dendrogram indicate samples from the ITF (blue) or from inner tumor (pink). Hierarchical clustering was performed in the R environment using Euclidean distance and complete ligation for neoplastic island samples and Euclidean distance and average ligation for tumor stroma samples.

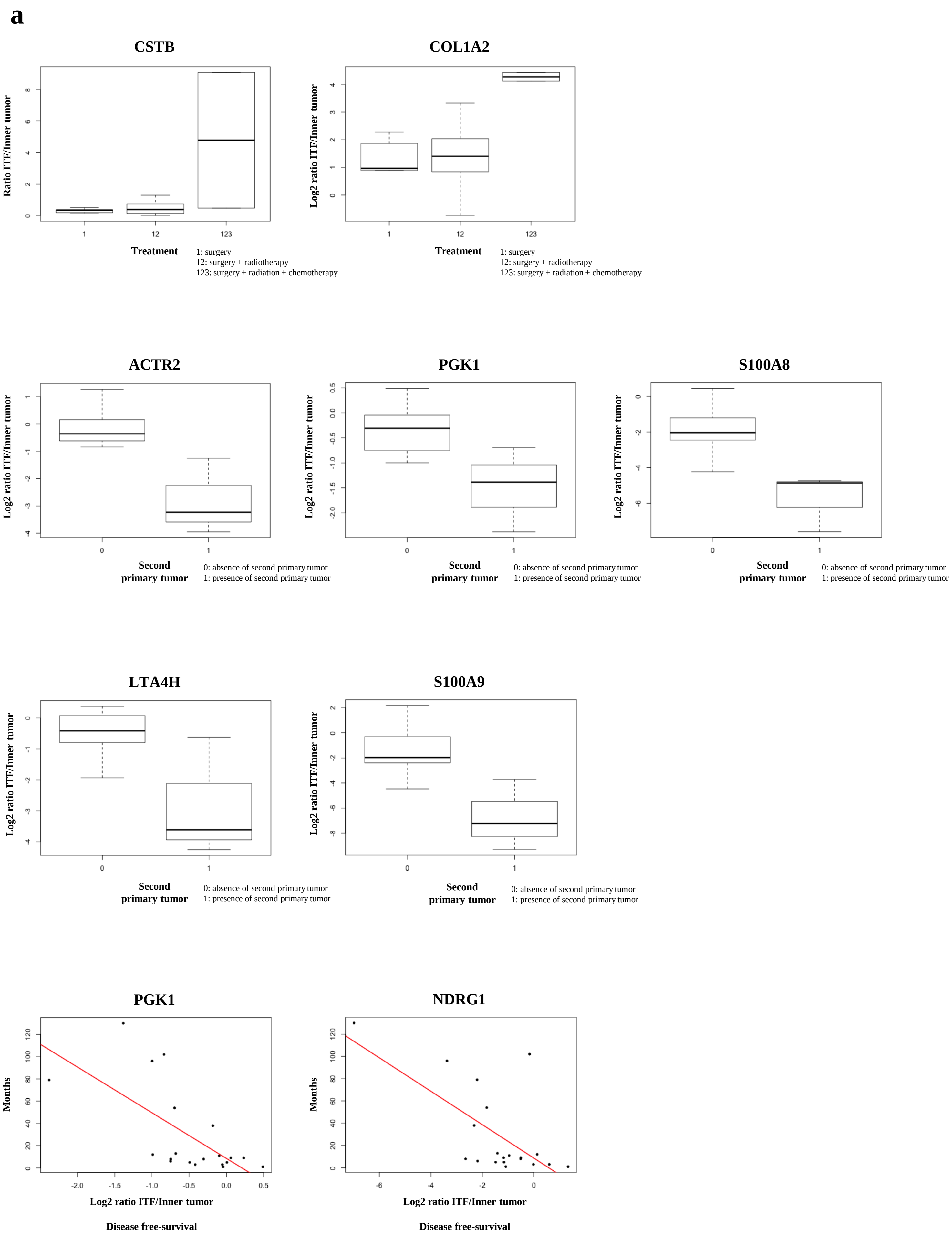
a
CSTB
Ratio ITF/Inner tumor
Treatment
1: surgery
12: surgery + radiotherapy
123: surgery + radiation + chemotherapy
COL1A2
Log2 ratio ITF/Inner tumor
Treatment
1: surgery
12: surgery + radiotherapy
123: surgery + radiation + chemotherapy
ACTR2
Log2 ratio ITF/Inner tumor
Second primary tumor
0: absence of second primary tumor
1: presence of second primary tumor
PGK1
Log2 ratio ITF/Inner tumor
Second primary tumor
0: absence of second primary tumor
1: presence of second primary tumor
S100A8
Log2 ratio ITF/Inner tumor
Second primary tumor
0: absence of second primary tumor
1: presence of second primary tumor
LTA4H
Log2 ratio ITF/Inner tumor
Second primary tumor
0: absence of second primary tumor
1: presence of second primary tumor
S100A9
Log2 ratio ITF/Inner tumor
Second primary tumor
0: absence of second primary tumor
1: presence of second primary tumor
PGK1
Months
Log2 ratio ITF/Inner tumor
Disease free-survival
NDRG1
Months
Log2 ratio ITF/Inner tumor
Disease free-survival

b

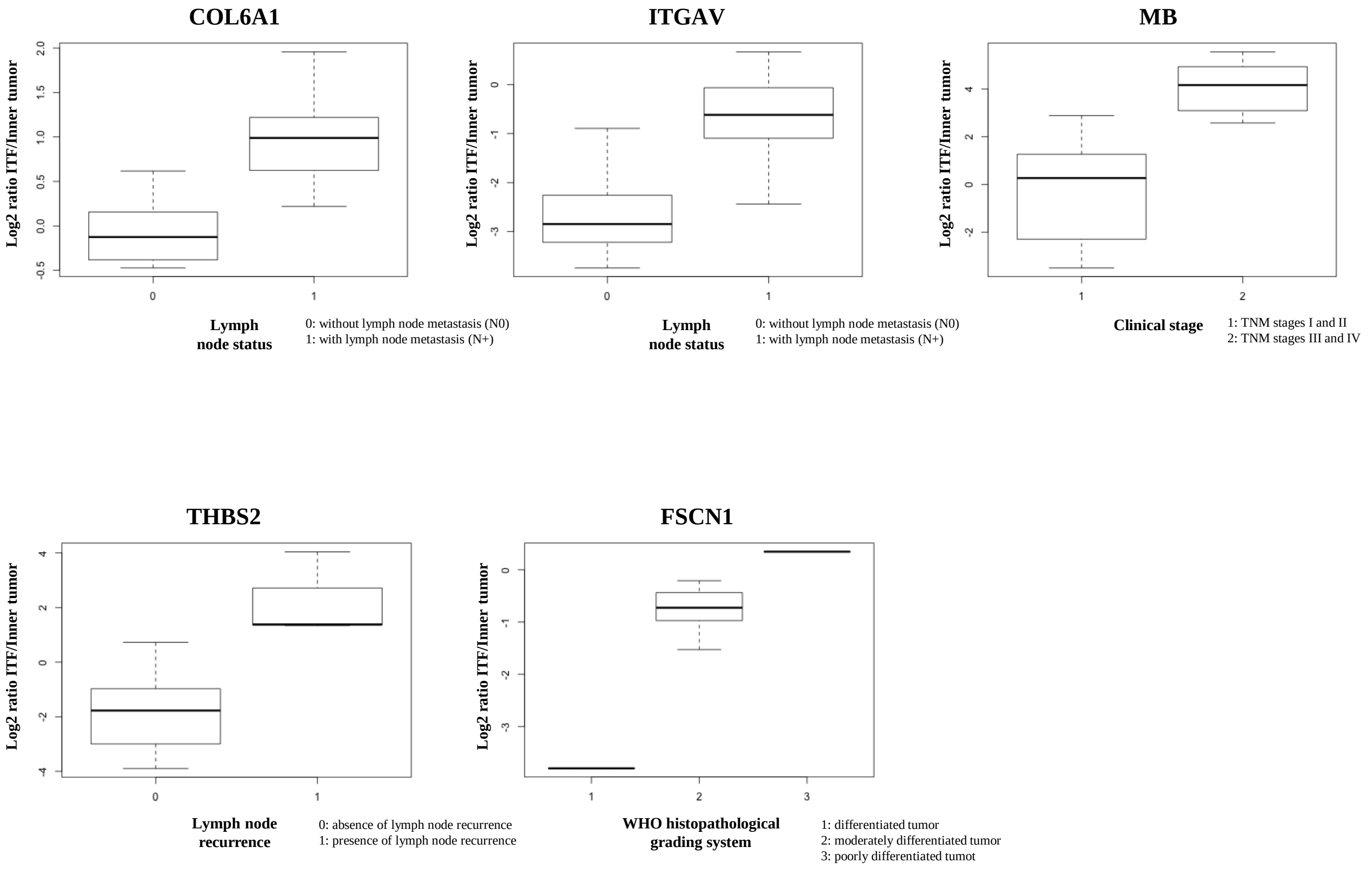

Supplementary Figure 4. Correlation plots of protein abundance and clinicopathological data. (a) Neoplastic islands proteins were associated to second primary tumor, treatment and disease free survival. (b) Stroma tumor proteins were associated to lymph node status, poorly differentiated tumor, lymph node recurrence and clinical stage. All box plots represent the median and interquartile range, whiskers represent the 1–99 percentile.

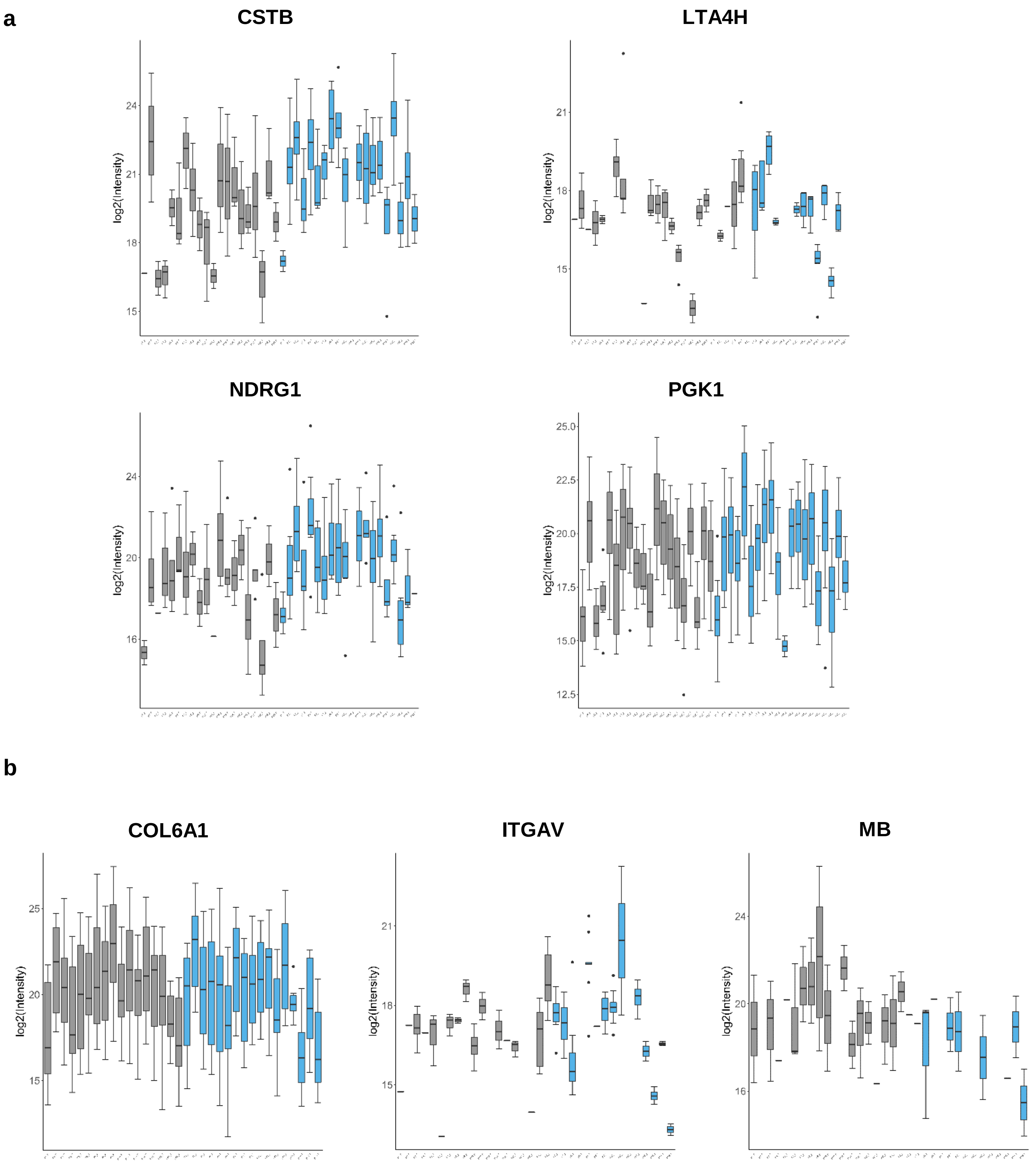

Supplementary Figure 5. Box plots of peptide intensities for the selected proteins. Box plots depict the abundance profile of the proteins selected for the verification step across all MS runs from discovery proteomics analysis of (a) neoplastic island samples and (b) tumor stroma samples. The distribution over each sample is represented by the intensity values of the identified peptides. Samples from the ITF are represented by gray color and samples from the inner tumor are indicated by blue color. All box plots represent the median and interquartile range, whiskers represent the 1–99 percentile, and outliers are represented by black dots.

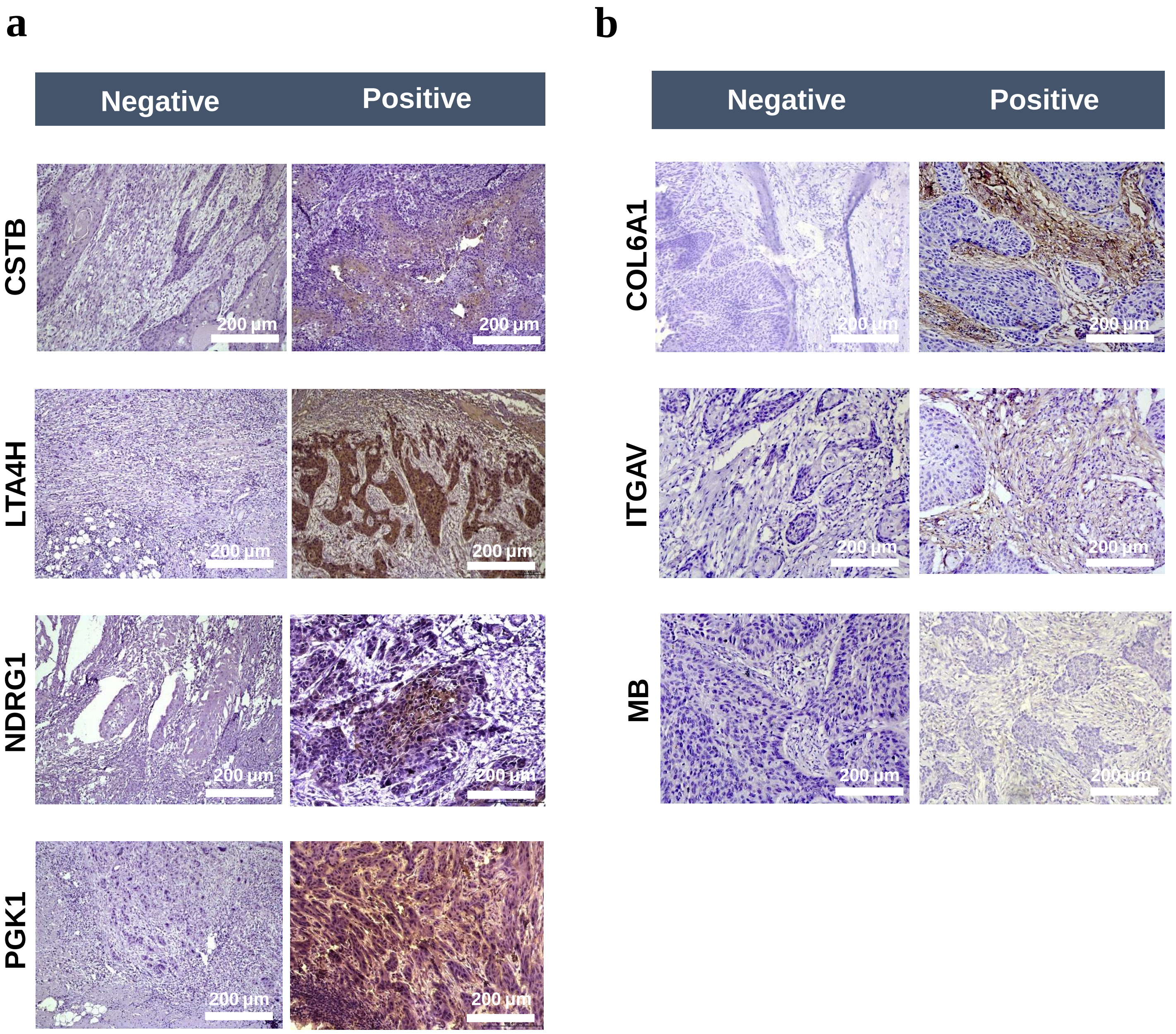

Supplementary Figure 6. Immunohistochemical staining of targeted proteins in OSCC tissues. Oral SCC tissue samples from a set of 125 cases were used to verify the abundance of (a) CSTB, LTA4H, NDRG1 and PGK1 neoplastic island proteins, and 96 cases were used to verify the abundance of (b) COL6A1, ITGAV and MB tumor stromal proteins. Among the cases, it was possible to verify both positive and negative staining. (Histological images were obtained using a 20X objective. Scale bars, 200 µm).

Cystatin B (CSTB)

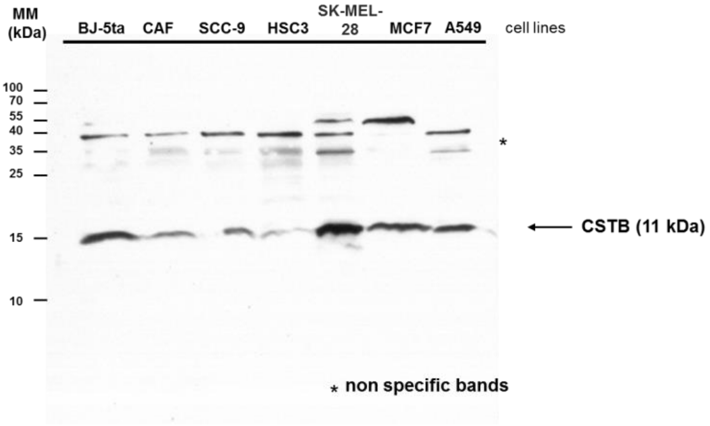

Leukotriene A-4 hydrolase (LTA4H)

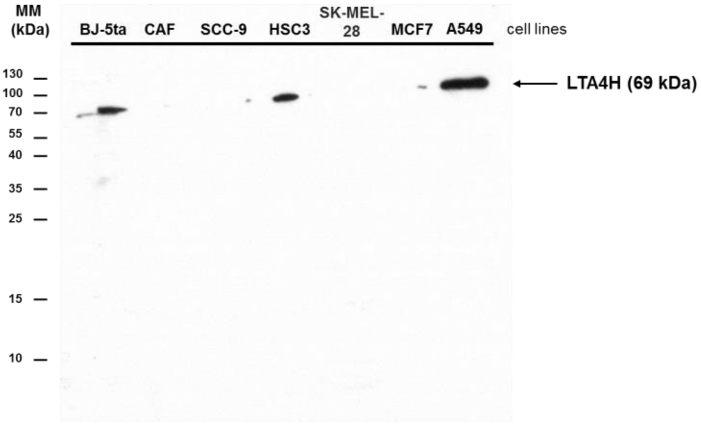

Protein NDRG1

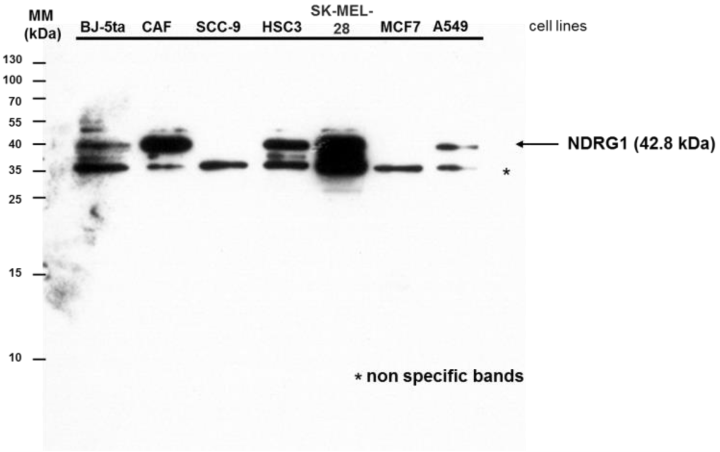

Phosphoglycerate kinase 1 (PGK1)

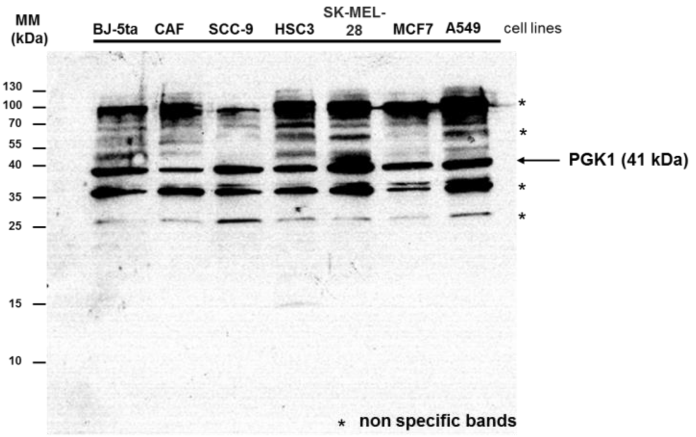

Collagen alpha-1(VI) chain (COL6A1)

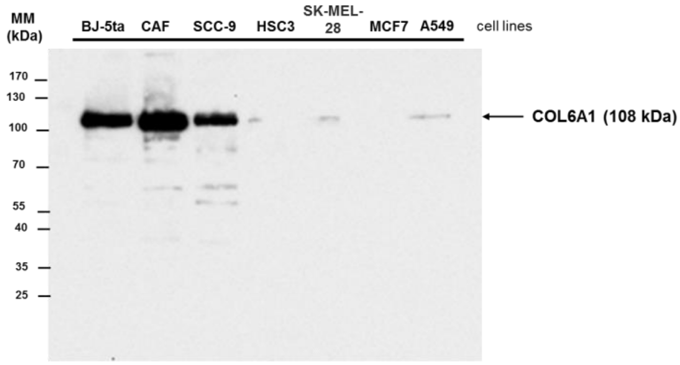

Integrin alpha-V (ITGAV)

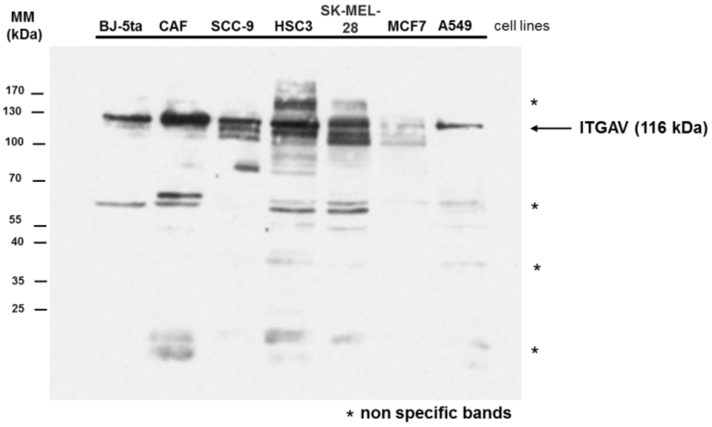

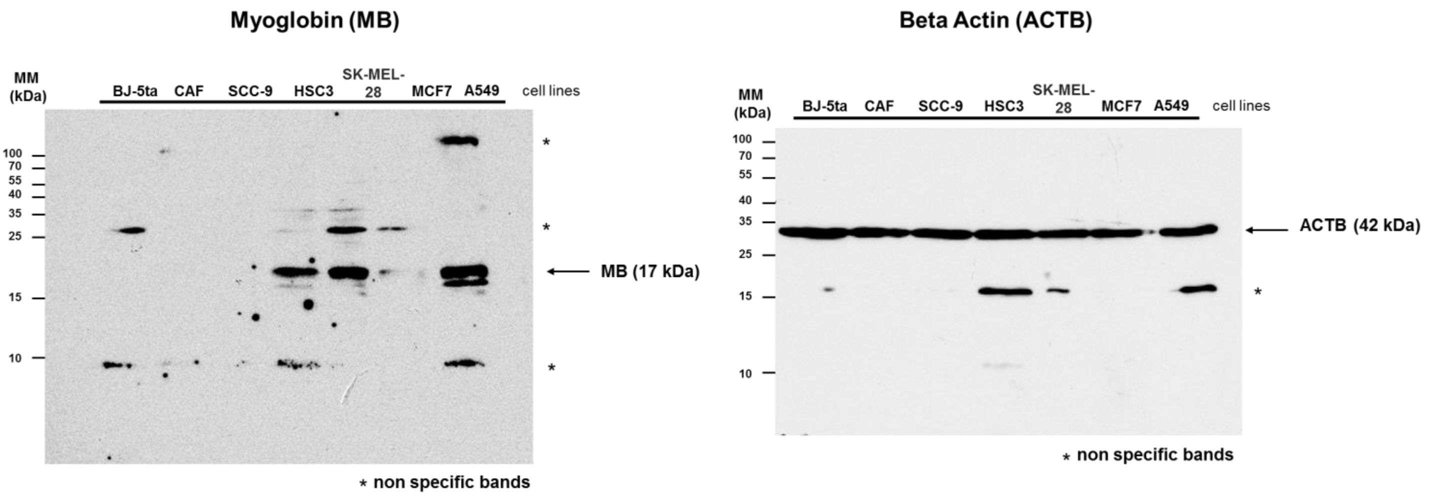

Supplementary Figure 7. Verification of specificity of antibodies used for IHC analysis. Forty micrograms of protein extracts derived from 7 cell lines (BJ-5ta ATCC CRL-4001, a fibroblast immortalized cell line; CAF, primary oral cancer associated fibroblasts; SCC-9 ATCC CRL-1629, a tongue cancer cell line; HSC3, a tongue cancer cell line; SK-MEL-28, a malignant skin-derived melanoma cell line; MCF7 ATCC HTB-22, a breast cancer cell line; and A549 ATCC CCL-185, an epithelial lung cancer cell line) were analyzed on a 10% SDS-PAGE and subjected to western blot with the same antibodies used for IHC analysis.

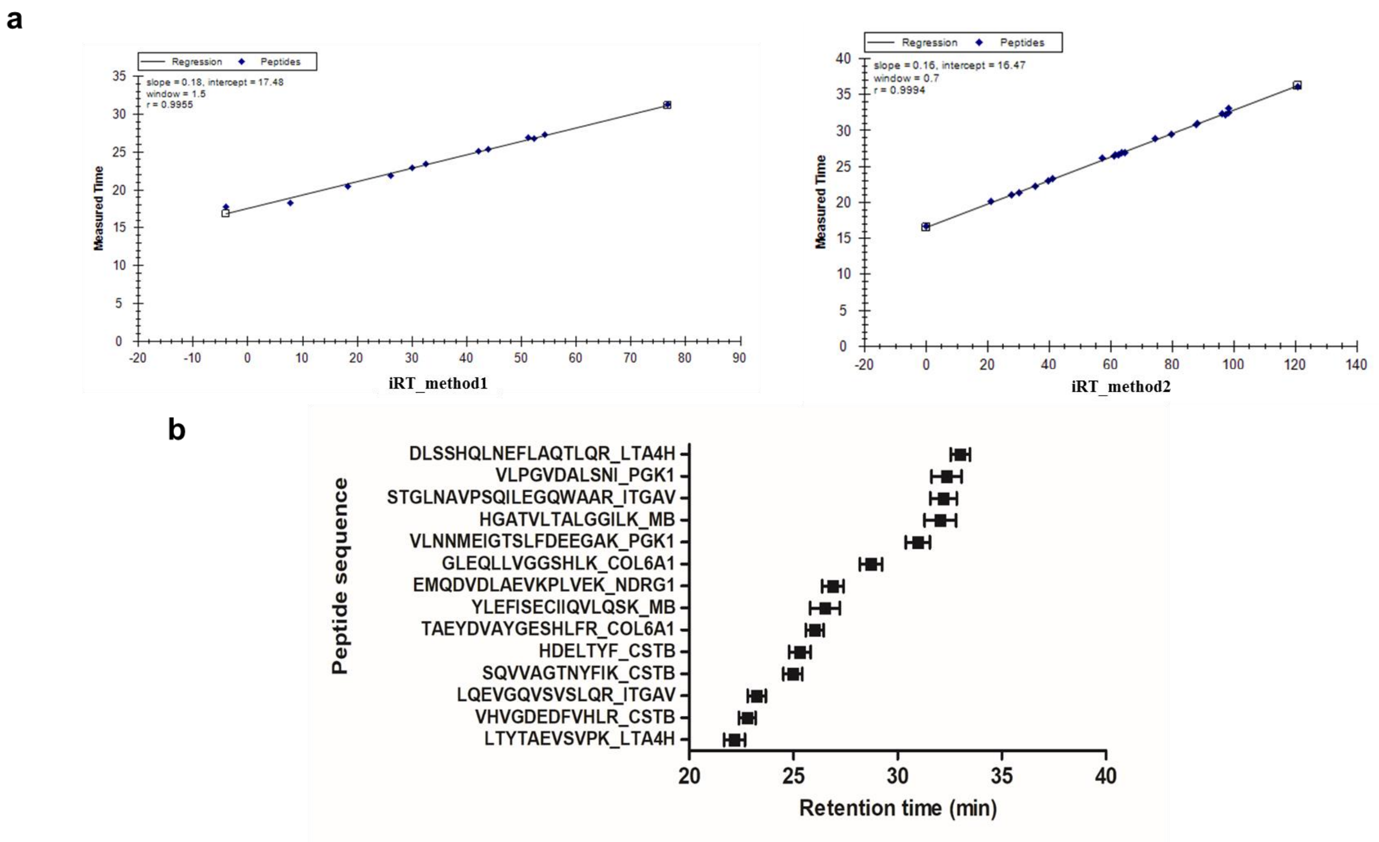

Supplementary Figure 8. Chromatographic retention time of the individual peptides in saliva analyzed by SRM assay. (A) Linear regression graph from Skyline shows that most peptides correlate with the prediction time. (B) The 14 peptides quantified by SRM in all cohort of saliva samples (40 samples analyzed in triplicates) demonstrate reproducibility in terms of retention time. All monitored peptides were detected and presented a measured retention time close to the predicted retention time among the replicates (r=0.9955 for set 1 and r=0.9994 for set 2). Error bars represent mean ± s.d..

COL6A1 - K.TAEYDVAYGESHLF**R**.V [990, 1004] – **N0**

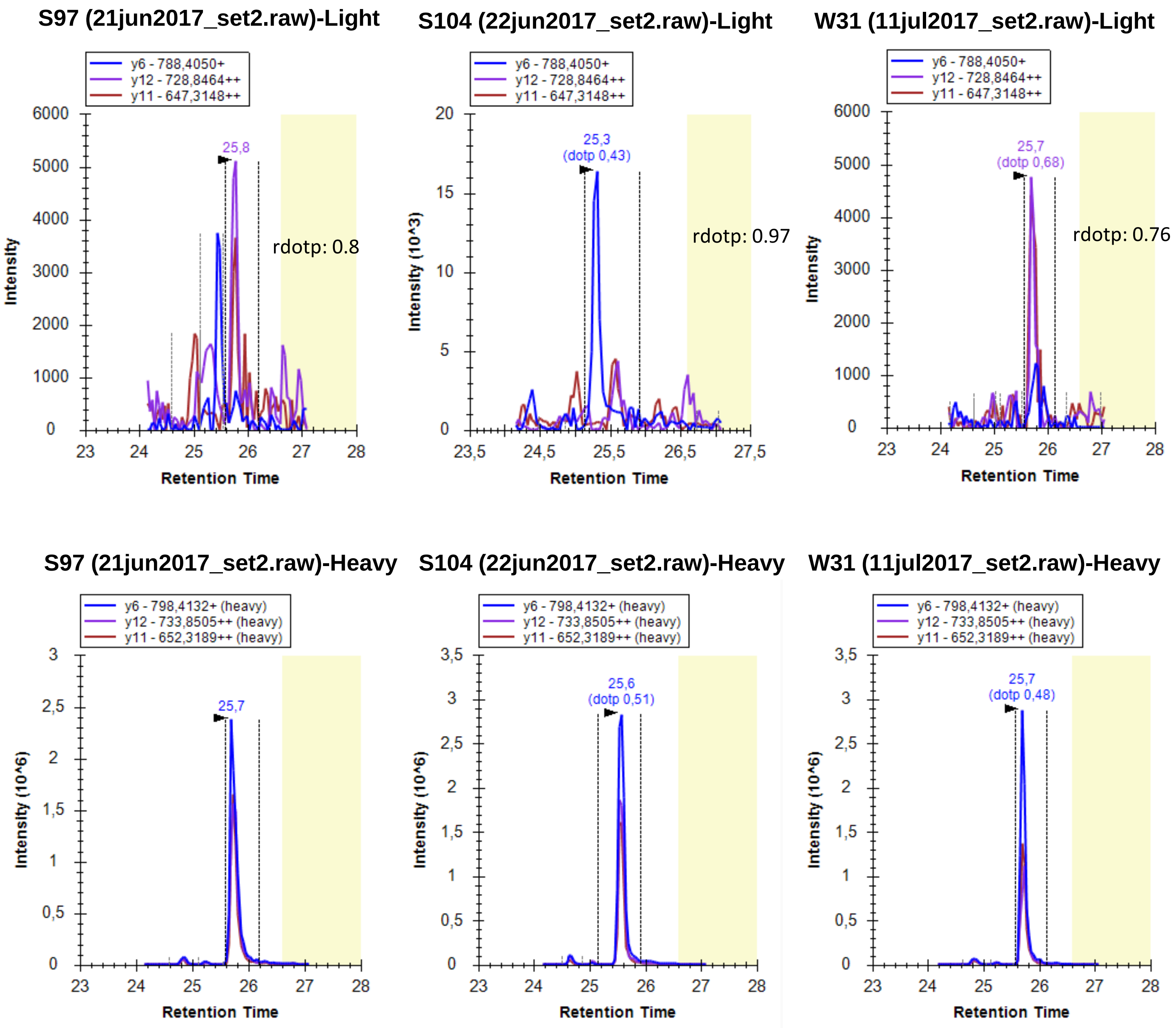

COL6A1 - K.TAEYDVAYGESHLF**R**.V [990, 1004] – **N+**

S106 (04jul2017_set2.raw)-Light

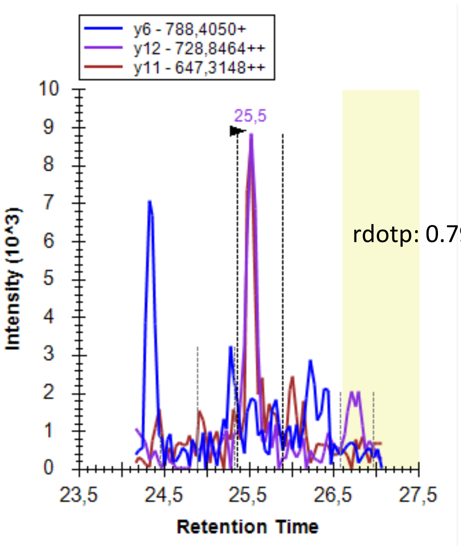

S44 (10jul2017_set2.raw)-Light

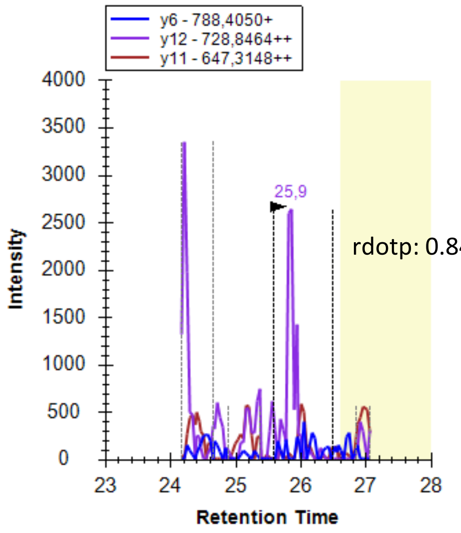

S82 (23jun2017_set2.raw)-Light

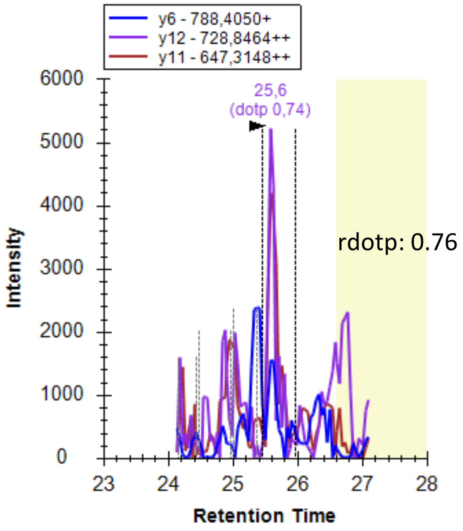

S97 (01jul2017_set2.raw)-Light

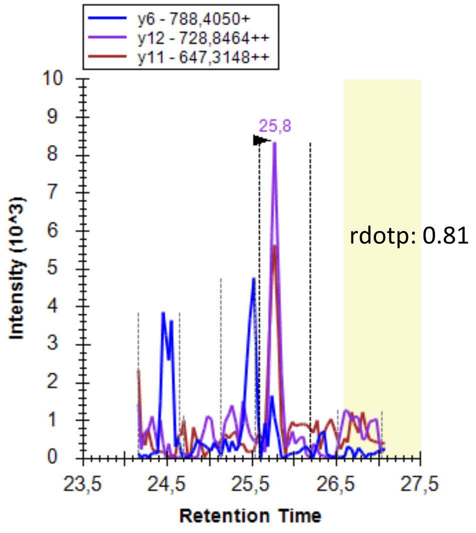

S106 (04jul2017_set2.raw)-Heavy

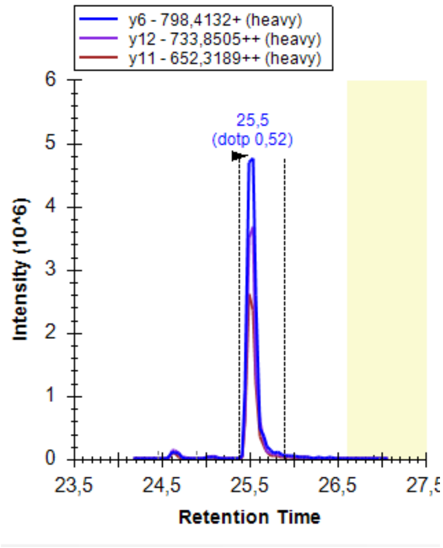

S44 (10jul2017_set2.raw)-Heavy

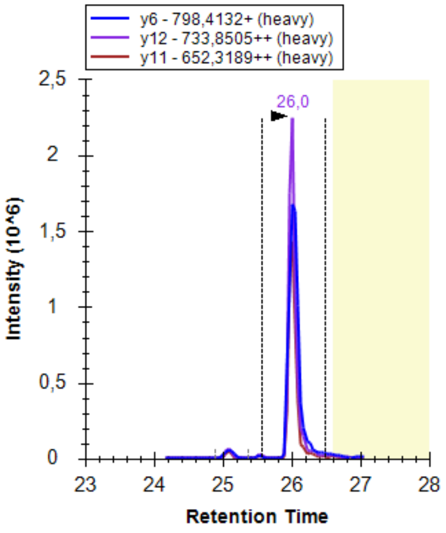

S82 (23jun2017_set2.raw)-Heavy

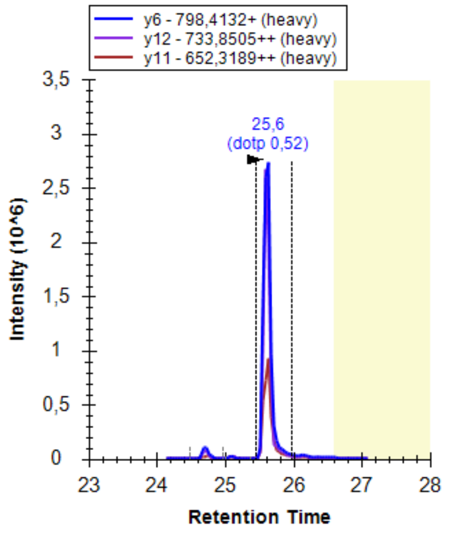

S97 (01jul2017_set2.raw)-Heavy

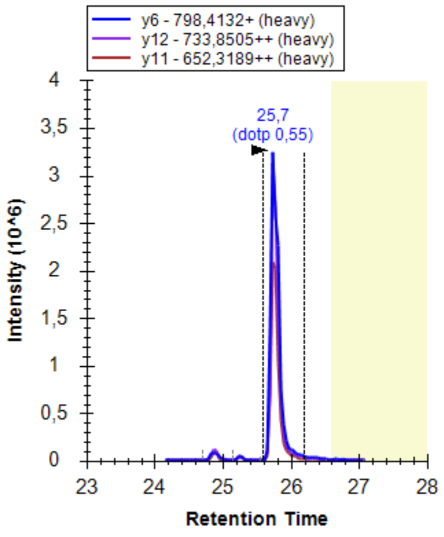

COL6A1 - K.GLEQLLVGGSHL**K**.E [135, 147] – **N0**

S110 (20jun2017_set2.raw)-Light

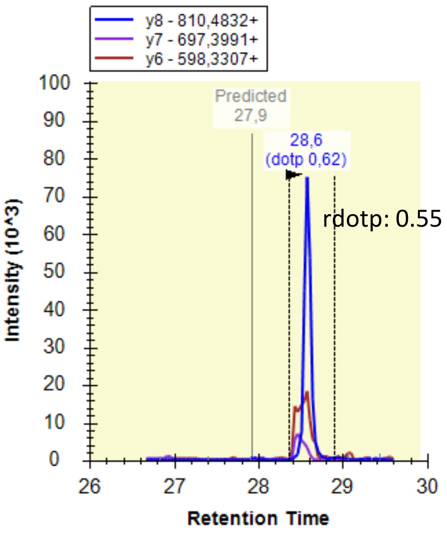

T2 (24jun2017_set2.raw)-Light

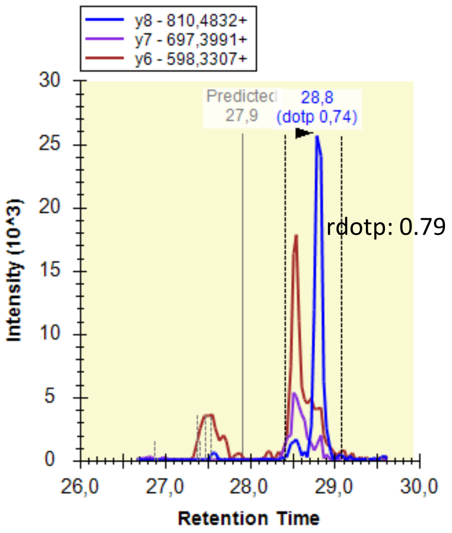

S120 (03jul2017_set2.raw)-Light

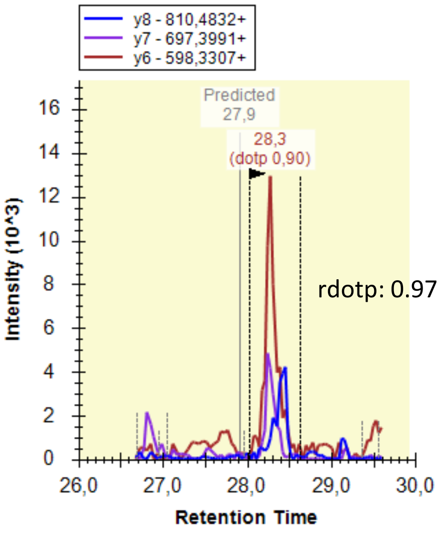

W31 (11jul2017_set2.raw)-Light

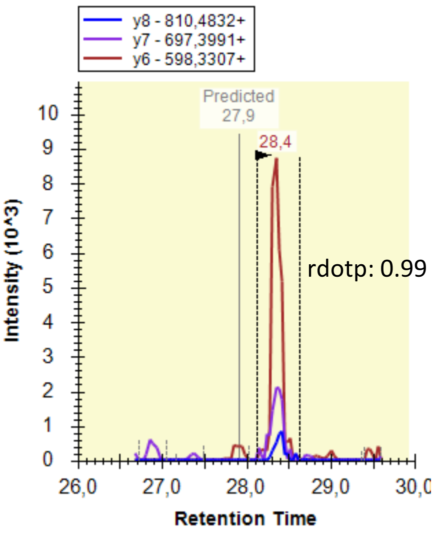

S110 (20jun2017_set2.raw)-Heavy

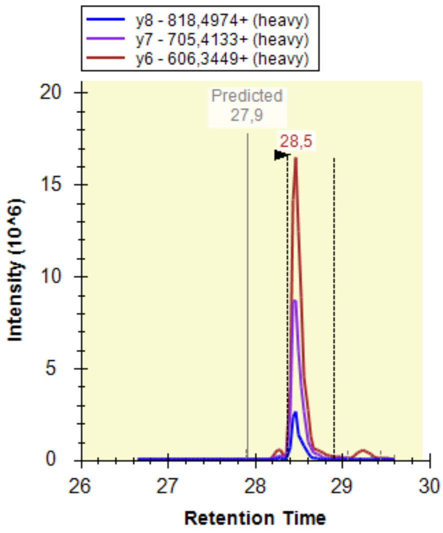

T2 (24jun2017_set2.raw)-Heavy

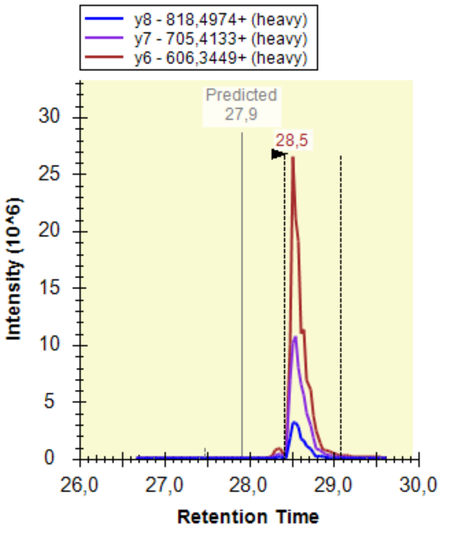

S120 (03jul2017_set2.raw)-Heavy

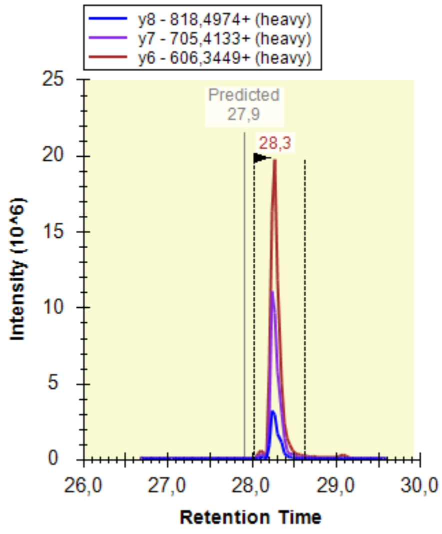

W31 (11jul2017_set2.raw)-Heavy

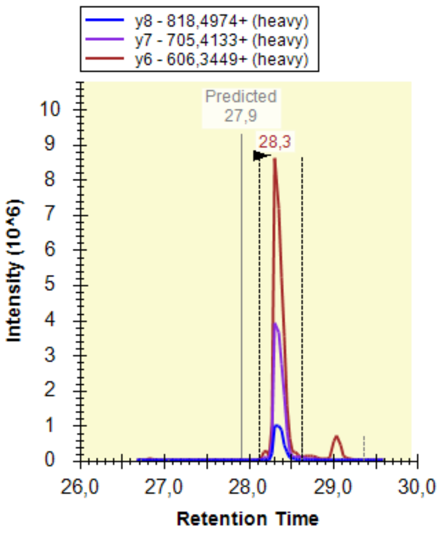

COL6A1 - K.GLEQLLVGGSHL**K.**E [135, 147] – **N+**

S77 (12jul_set2.raw)-Light

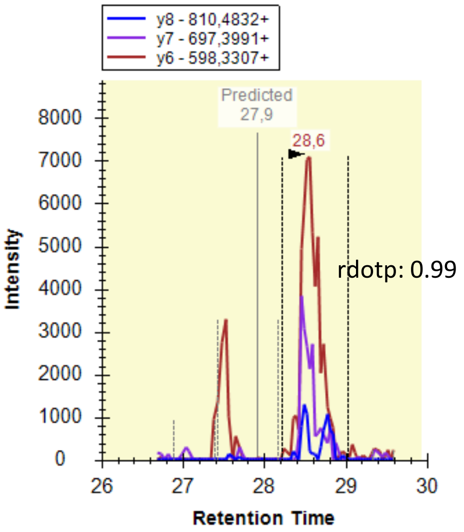

S32 (14jul_set2.raw)-Light

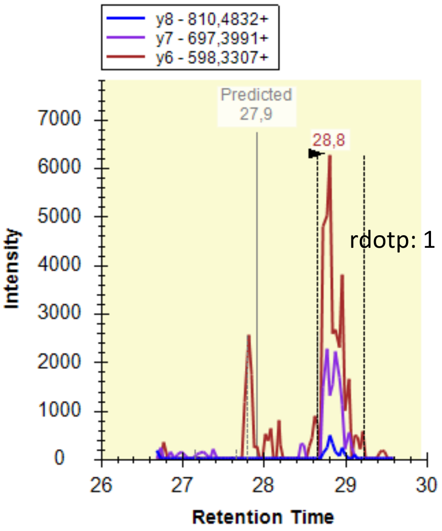

S12 (13jul_set2.raw)-Light

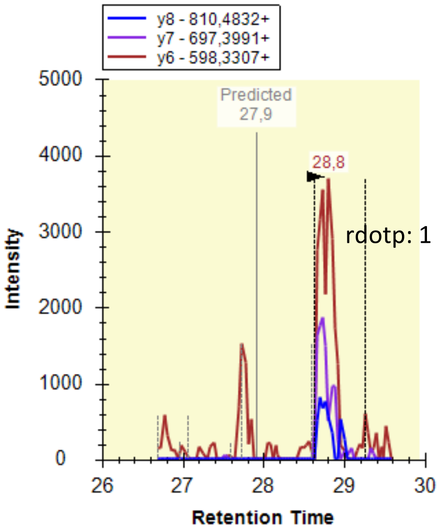

S116 (22jun_set2.raw)-Light

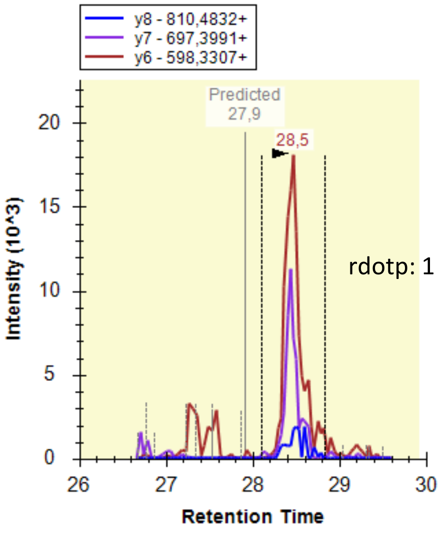

S77 (12jul_set2.raw)-Heavy

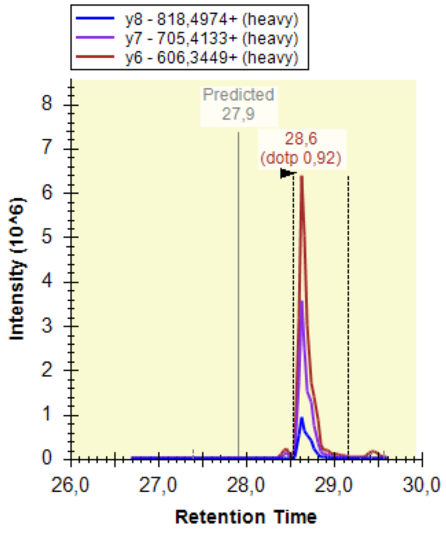

S32 (14jul_set2.raw)-Heavy

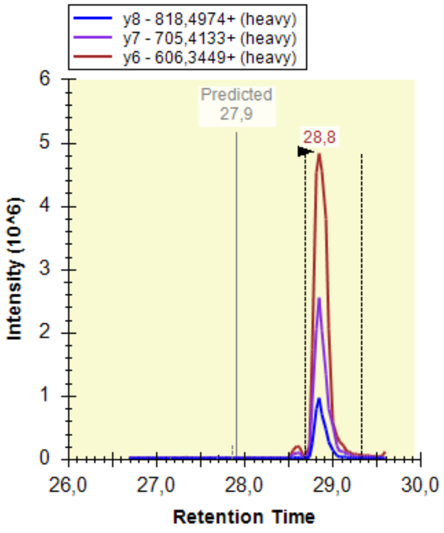

S12 (13jul_set2.raw)-Heavy

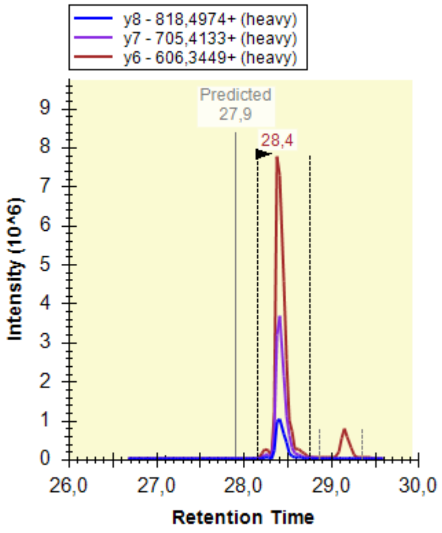

S116 (22jun_set2.raw)-Heavy

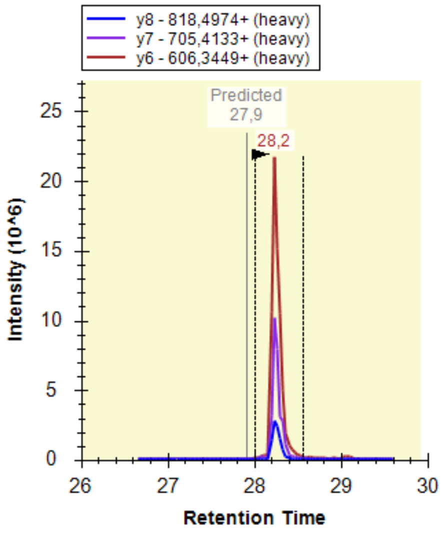

LTA4H - K.DLSSHQLNEFLAQTL**QR**.A [493, 509] – **N0**

T2 (24jun_set2.raw)-Light

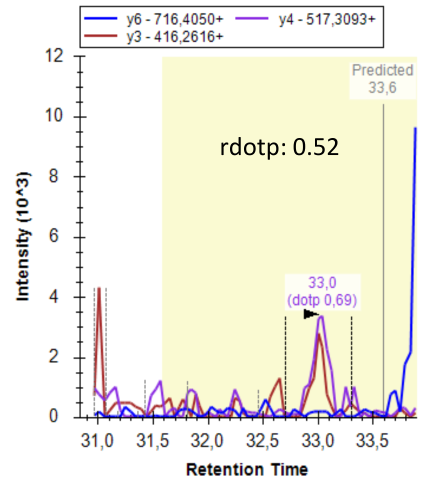

W31 (24jun_set2.raw)-Light

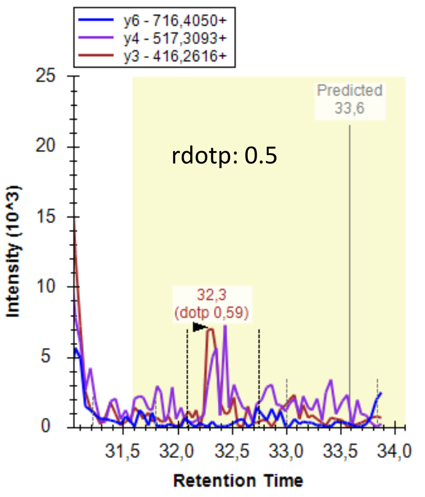

W32 (23jun_set2.raw)-Light

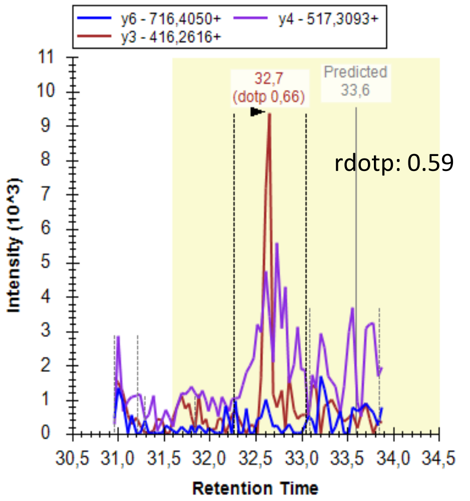

W32 (29jun_set2.raw)-Light

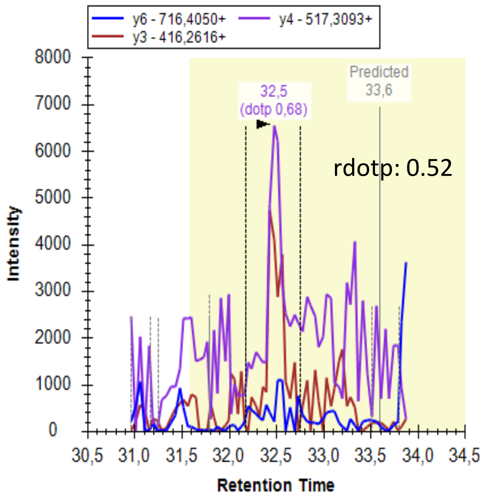

W31 (11jul_set2.raw)-Light

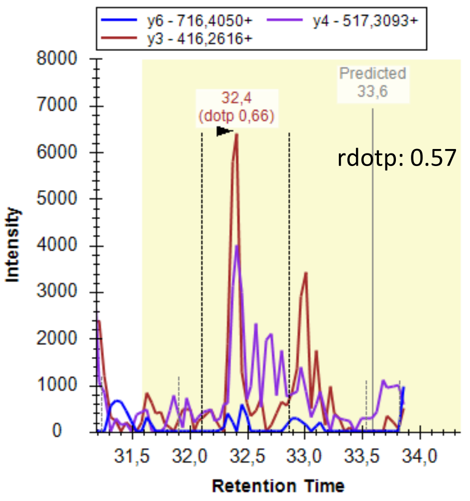

T2 (24jun_set2.raw)-Heavy

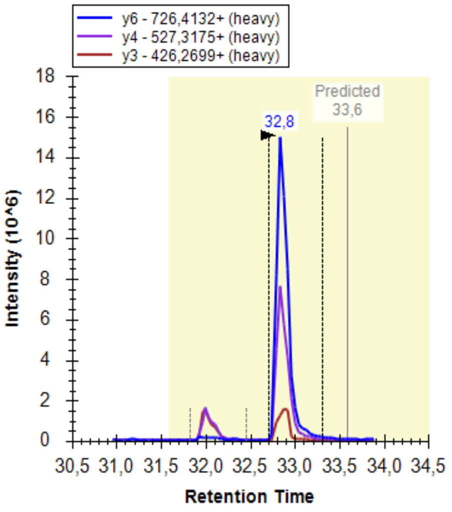

W31 (24jun_set2.raw)-Heavy

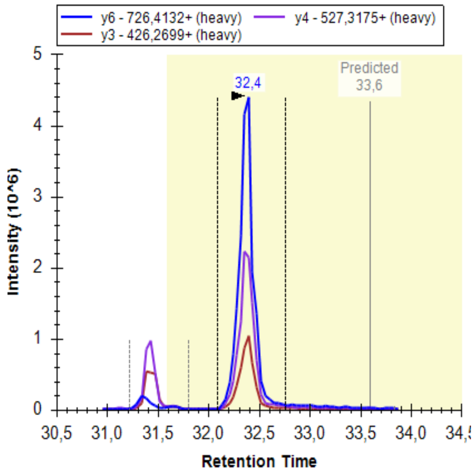

W32 (23jun_set2.raw)-Heavy

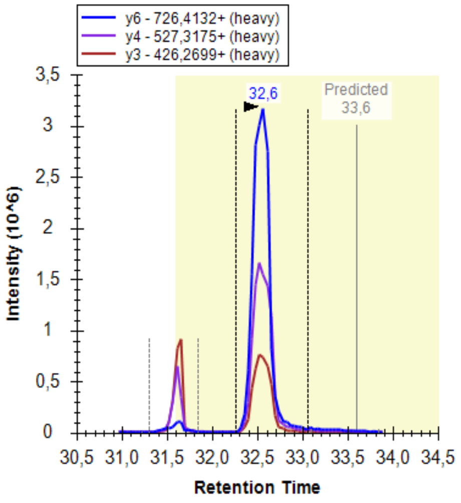

W32 (29jun_set2.raw)-Heavy

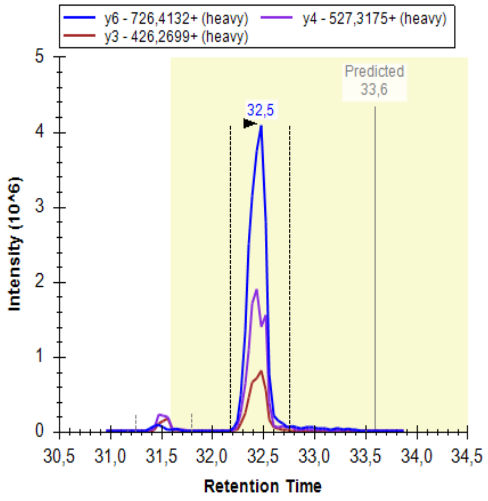

W31 (11jul_set2.raw)-Heavy

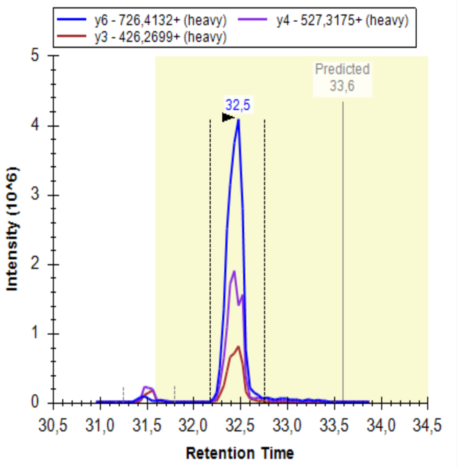

LTA4H - K.DLSSHQLNEFLAQTLQ**R**.A [493, 509] – **N+**

S82 (13jul_set2.raw)-Light

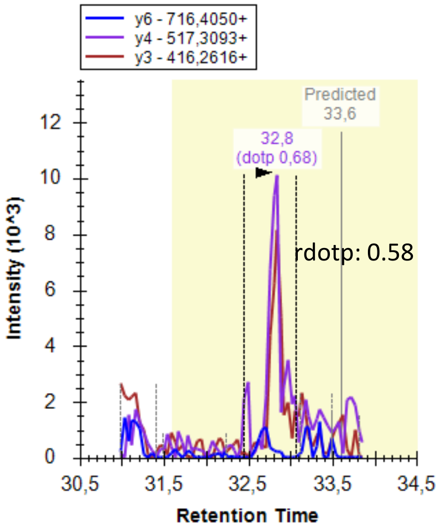

S69 (08jul_set2.raw)-Light

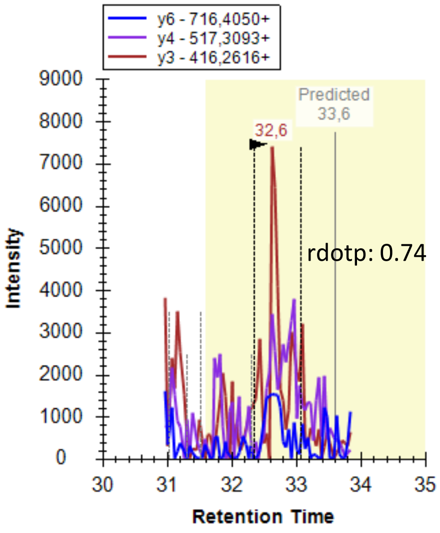

S12 (06jul_set2.raw)-Light

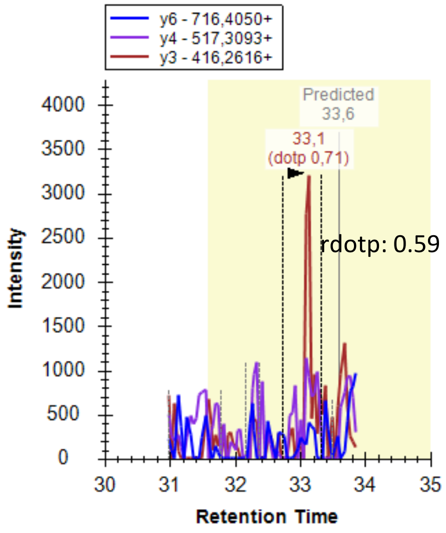

S82 (23jun_set2.raw)-Light

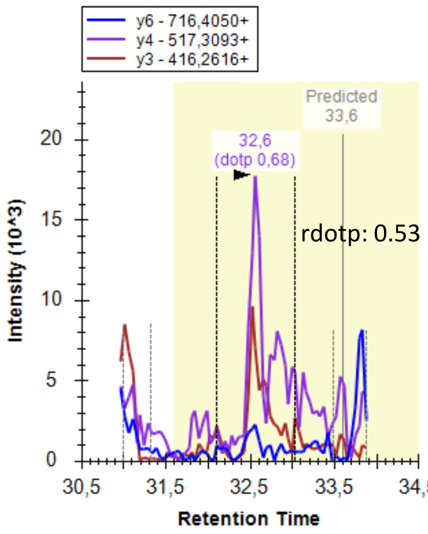

S82 (13jul_set2.raw)-Heavy

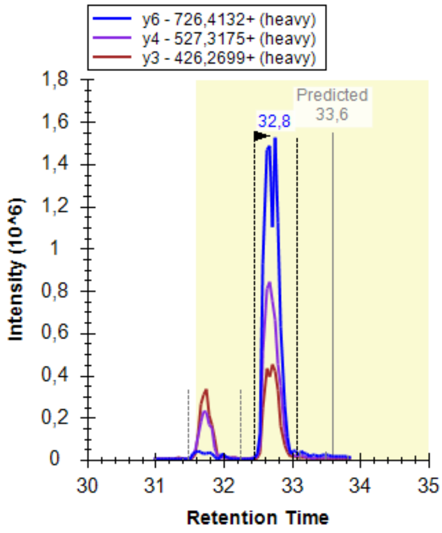

S69 (08jul_set2.raw)-Heavy

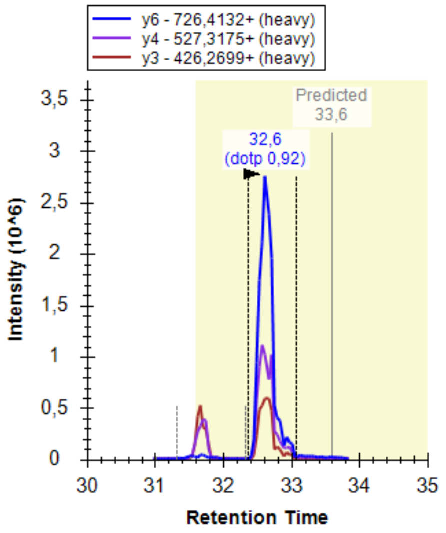

S12 (06jul_set2.raw)-Heavy

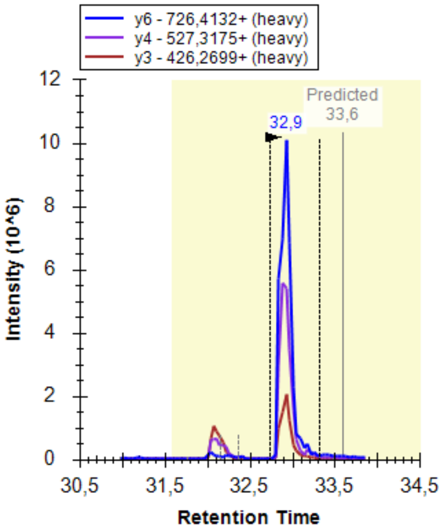

S82 (23jun_set2.raw)-Heavy

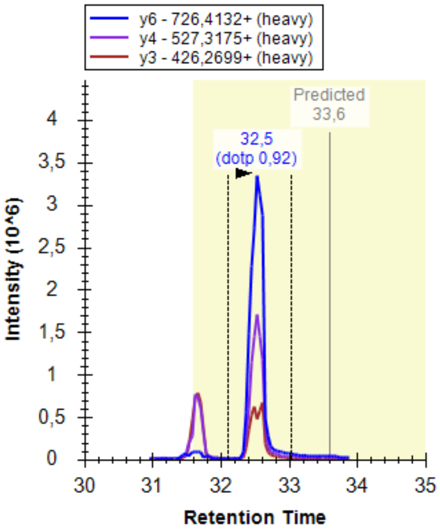

LTA4H - K.LTYTAEVSVP**K**.E [154, 164] – **N0**

S120 (03jul_set2.raw)-Light

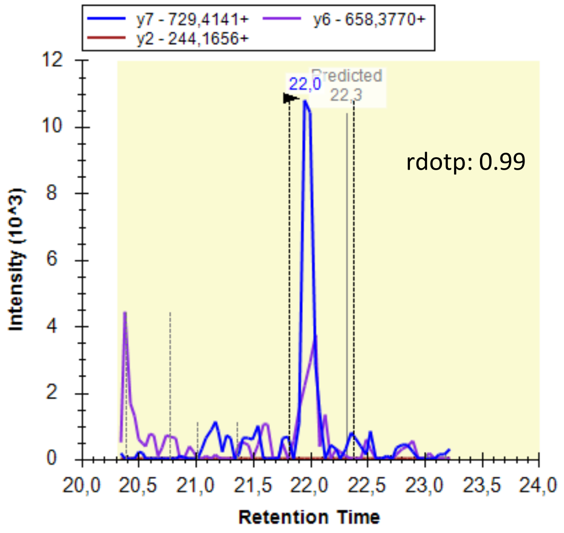

S110 (03jul_set2.raw)-Light

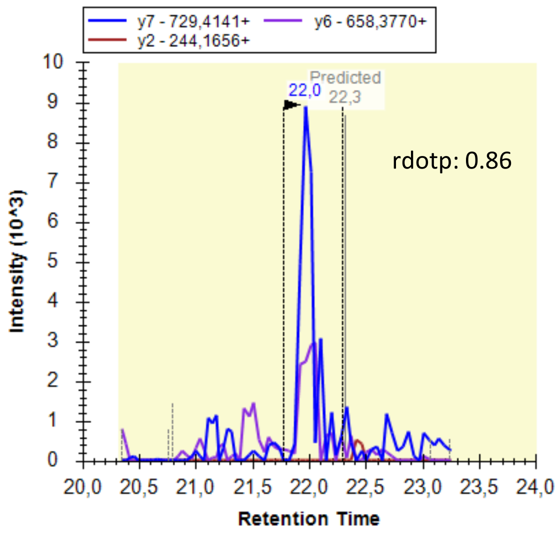

W32 (03jul_set2.raw)-Light

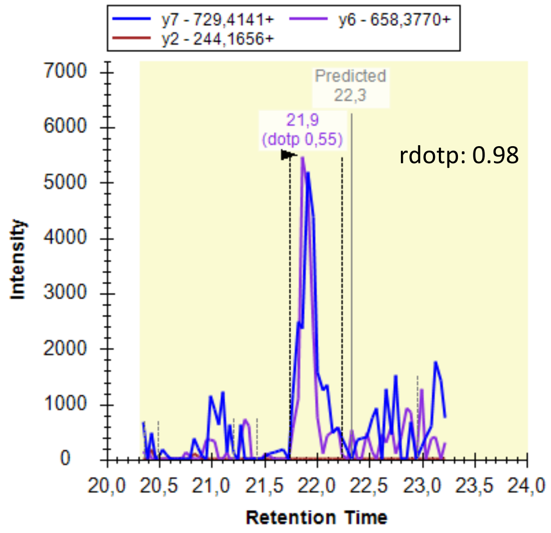

S120 (03jul_set2.raw)-Heavy

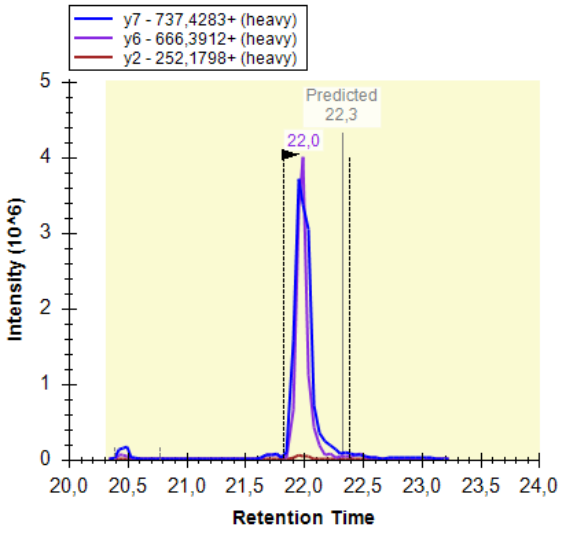

S110 (03jul_set2.raw)-Heavy

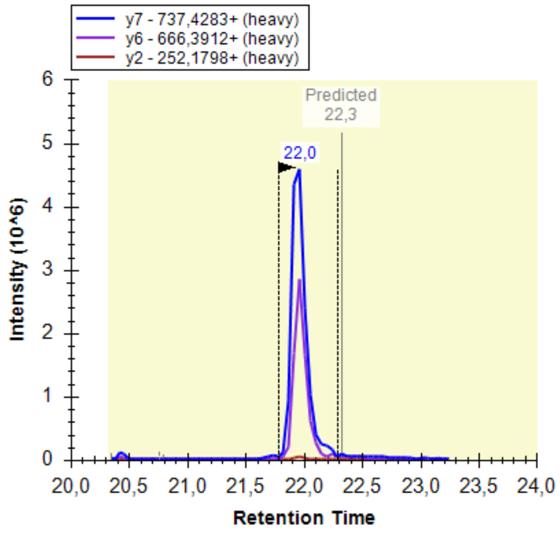

W32 (03jul_set2.raw)-Heavy

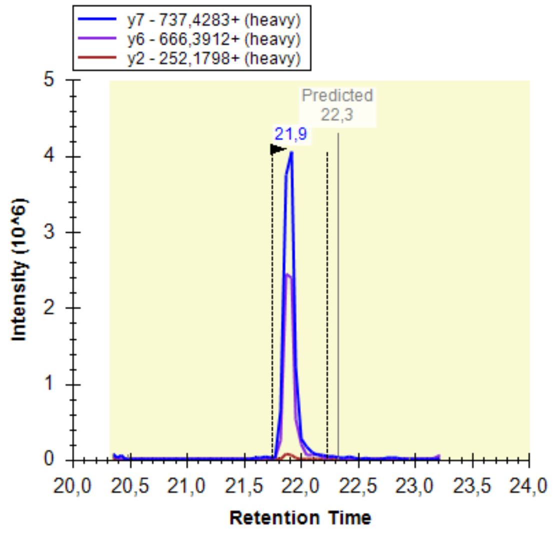

LTA4H - K.LTYTAEVSVP**K**.E [154, 164] – **N+**

S67 (24jun_set2.raw)-Light

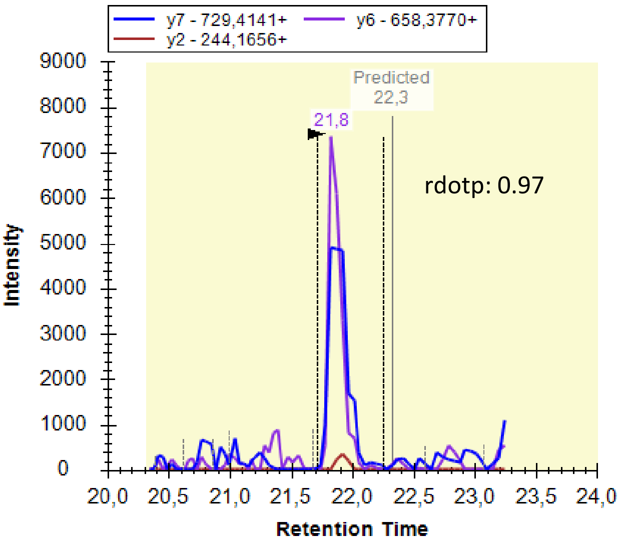

S77 (24jun_set2.raw)-Light

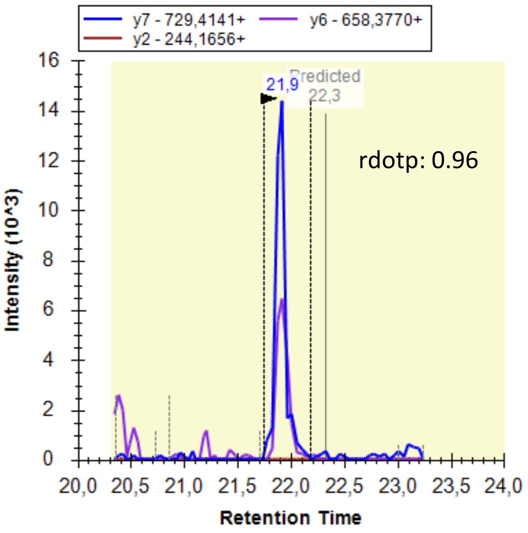

S12 (24jun_set2.raw)-Light

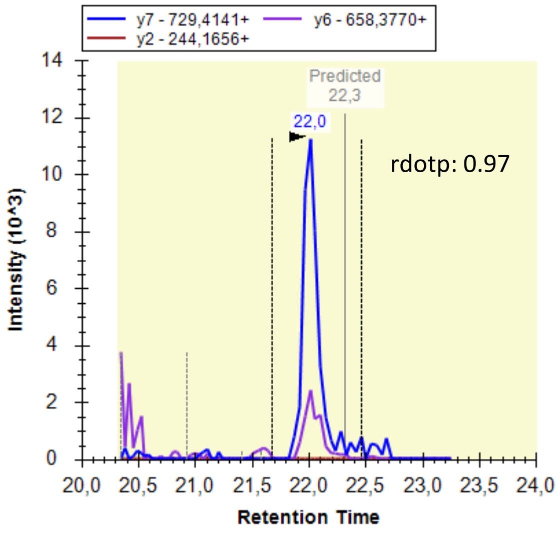

S67 (24jun_set2.raw)-Heavy

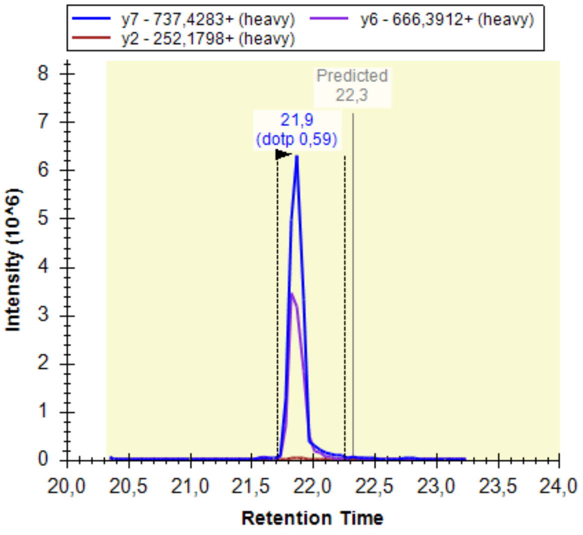

S77 (24jun_set2.raw)-Heavy

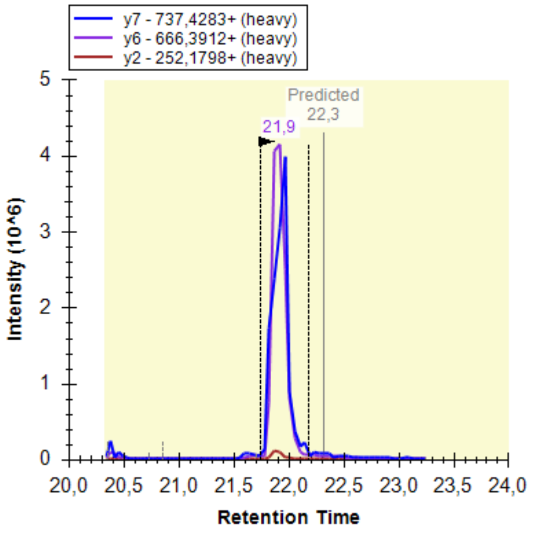

S12 (24jun_set2.raw)-Heavy

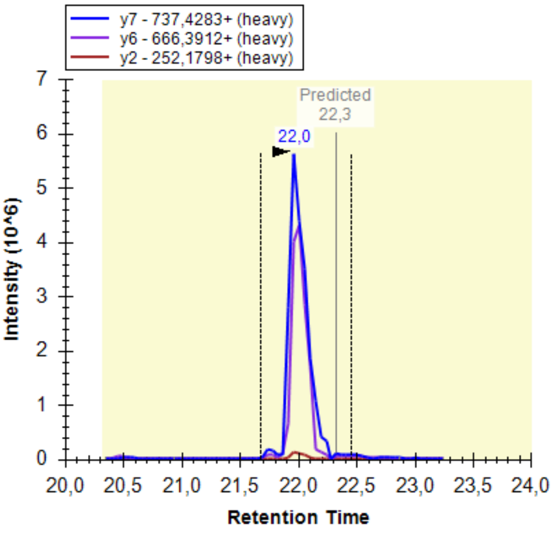

Supplementary Figure 9. Chromatograms of proteotypic peptides of LTA4H and COL6A1. The complete data are available in Panorama [https://panoramaweb.org/labkey/saliva_SRM.url].

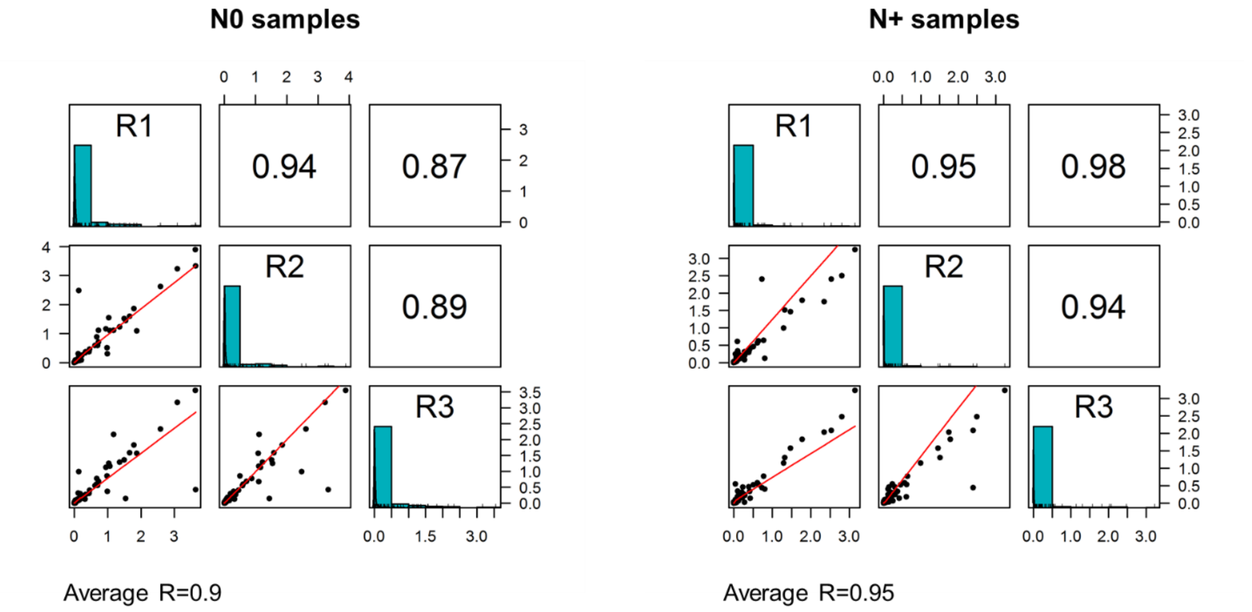

Supplementary Figure 10. Correlation plots of saliva technical replicate samples (N0 and N+). Representation of the reproducibility by Pearson's correlation of saliva triplicate samples for all 14 peptides (Log2 L/H ratio) stratified by patient group (N0 and N+ samples). Triplicates demonstrated strong reproducibility among the Log2 L/H ratio obtained for 14 peptides, with high Pearson correlation coefficients in the conditions evaluated, N0 (R>0.87) and N+ (R>0.94) samples.

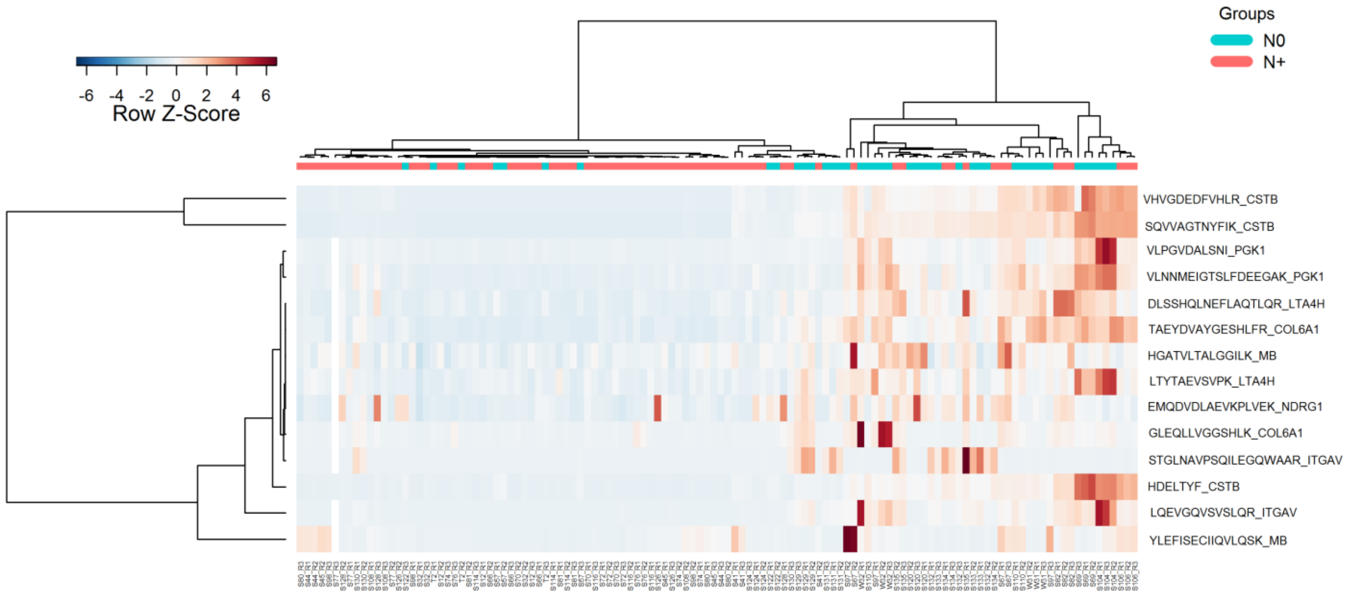

Supplementary Figure 11. Unsupervised hierarchical clustering analysis of peptides quantified in the saliva technical replicates segregated the samples in two main groups based on the lymph node status of the OSCC patients, N+ (presence of lymph node metastasis) and N0 (absence of lymph node metastasis). Values for each peptide (14 peptides; rows) and for each saliva replicate sample (239 MS runs; columns) are colored based on the peptide abundance, in which high (red) and low (blue) values (Z-scored log2 L/H ratio) are indicated in the color scale bar at the top right. The colored bars above the graph indicate samples from N0 (blue) or from N+ (pink). Hierarchical clustering was performed in the R environment using Euclidean distance with complete ligation.

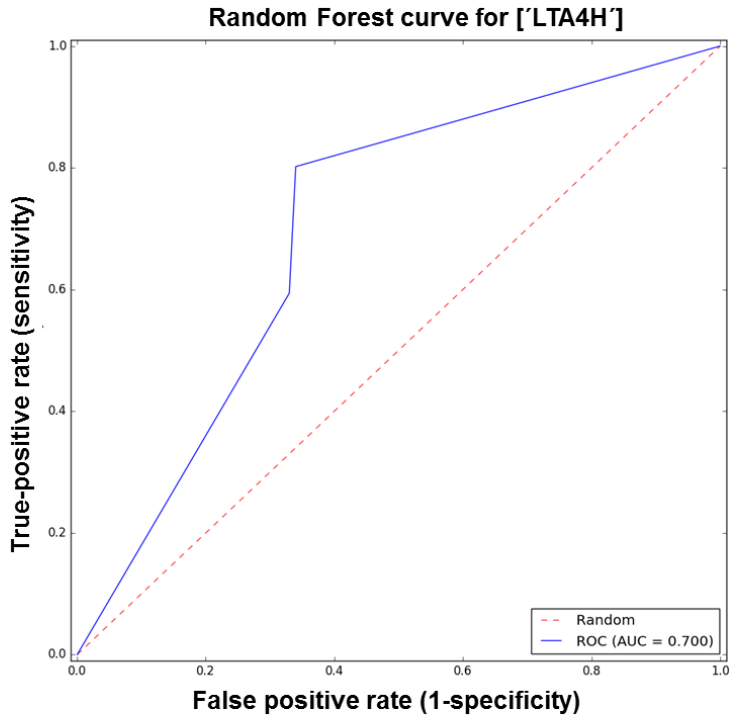

Supplementary Figure 12. ROC curves for independent test set analysis. The performance of the best protein signatures (LTA4H) is shown by the cross-validation ROC curve. The independent test set were composed by 20% of the patients in the dataset.

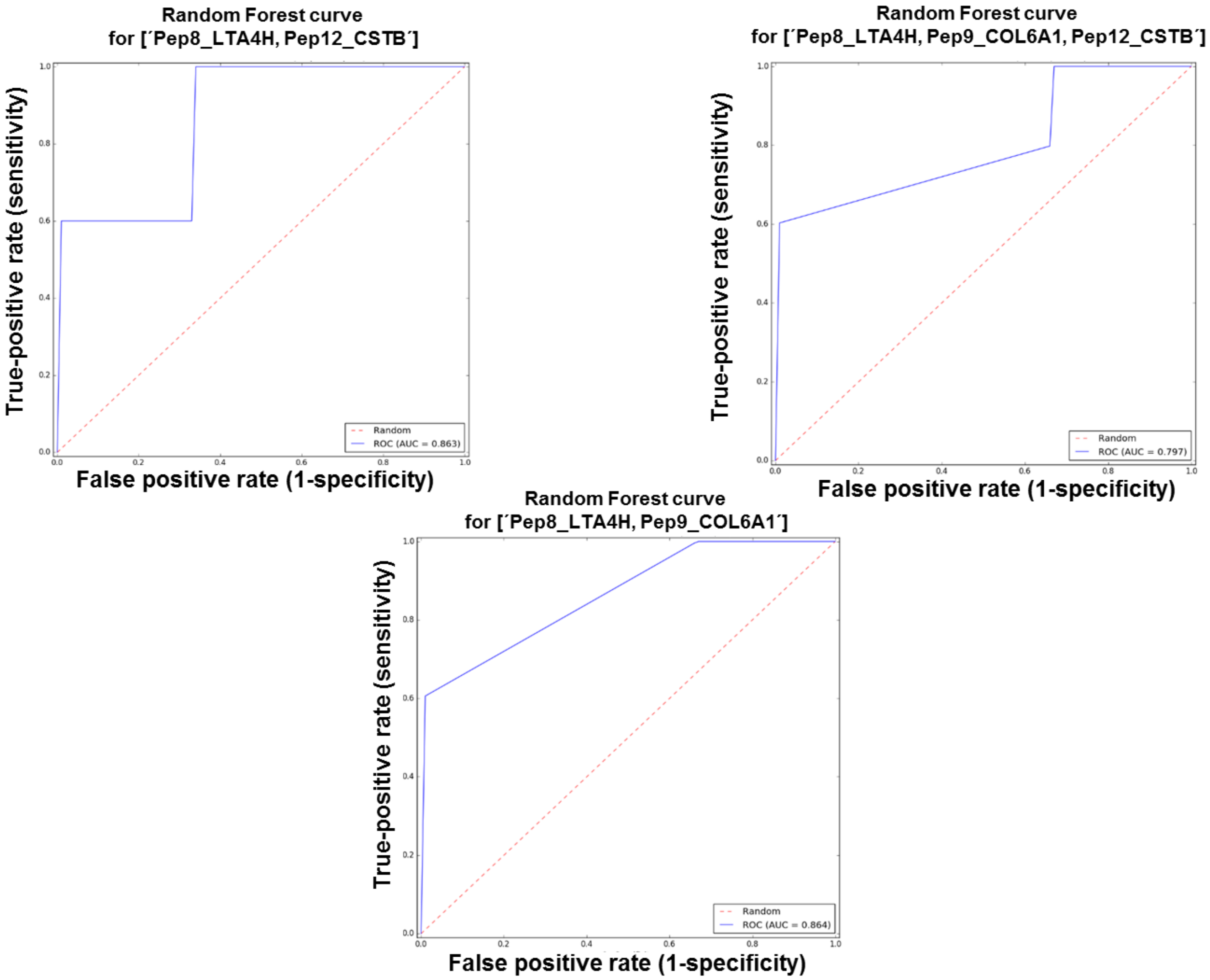

Supplementary Figure 13. ROC curves for independent test set analysis. The performance of the best peptide signatures is shown by the cross-validation ROC curve. The independent test set were composed by 20% of the patients in the dataset.
