# Supplementary material for: Combining discovery and targeted proteomics reveals a prognostic signature in oral cancer
Source: Nat Commun. 2018 Sep 5;9:3598. doi: 10.1038/s41467-018-05696-2 (PMC6125363; doi:10.1038/s41467-018-05696-2)
Supplement: Supplementary file 3 — Description of Additional Supplementary Files [file 41467_2018_5696_MOESM3_ESM.pdf]

Description of Additional Supplementary Files

File Name: Supplementary Data 1.
Description: Clinicopathological data of OSCC patient samples used for Discovery Proteomics.

File Name: Supplementary Data 2.
Description: Order of sample runs in the mass spectrometer for Discovery Proteomics.

File Name: Supplementary Data 3.
Description: Correlation plots of the neoplastic island samples from the Invasive Tumor Front (ITF) and from the inner tumor analyzed by discovery proteomics. Log2 LFQ intensity values of protein dataset after reverse and 'only' by site entries were used to calculate the Pearson correlation coefficient (indicated in blue) using Perseus software.

File Name: Supplementary Data 4.
Description: Correlation plot of the stroma tumor samples from the Invasive Tumor Front (ITF) and from the inner tumor analyzed by discovery 10 proteomics. Log2 LFQ intensity values of protein dataset after reverse and 'only' by site entries were used to calculate the Pearson correlation coefficient (indicated in blue) using Perseus software.

File Name: Supplementary Data 5.
Description: All proteins identified in neoplastic islands from ITF and from inner tumor by Discovery Proteomics, after excluding reverse sequences.

File Name: Supplementary Data 6.
Description: Proteins identified in neoplastic islands of the ITF and inner tumor by Discovery Proteomics, after excluding reverse sequences and 'only identified by site' entries.

File Name: Supplementary Data 7.
Description: Proteins identified in neoplastic islands from the ITF and inner tumor by Discovery Proteomics, after filtering by at least 10 valid values.

File Name: Supplementary Data 8.
Description: Differentially abundance proteins in neoplastic islands from ITF and inner tumor in Discovery Proteomics, after replacement of missing values by data imputation.

File Name: Supplementary Data 9.
Description: Front/Inner Ratio of differentially abundant proteins in neoplastic islands by Discovery Proteomics.

File Name: Supplementary Data 10.
Description: All identified proteins in tumor stroma by Discovery Proteomics, after excluding reverse sequences.

File Name: Supplementary Data 11.
Description: Proteins identified in tumor stroma by Discovery Proteomics, after excluding reverse sequences and 'only identified by site' entries.

File Name: Supplementary Data 12.
Description: Proteins identified in tumor stroma by discovery proteomics, after filtering by at least 8 valid values.

File Name: Supplementary Data 13.
Description: Differentially abundant proteins in front and inner tumor stroma by discovery proteomics, with replacement of missing values by data imputation.

File Name: Supplementary Data 14.
Description: Front/Inner Ratio of differentially expressed proteins in tumor stroma by discovery proteomics.

File Name: Supplementary Data 15.
Description: Enriched biological processes for proteins 'uniquely' identified in neoplastic island by discovery proteomics using the plugin BinGO within Cytoscape. - Supplementary Data 16. Enriched biological processes for proteins 'uniquely' identified in tumor stroma by discovery proteomics using the plugin BinGO within Cytoscape.

File Name: Supplementary Data 17.
Description: Enriched biological processes for differential proteins between neoplastic island and tumor stroma by discovery proteomics using the plugin BinGO within Cytoscape. Fold enrichment was calculated based on the 11 number of genes from the input that were annotated in Gene Ontology terms and the total number of genes in the input.

File Name: Supplementary Data 18.
Description: Criteria for priotirization of CSTB, LTA4H, NDRG1, PGK1, COL6A1, ITGAV and MB proteins, and information of their localization.

File Name: Supplementary Data 19.
Description: Clinicopathological data of OSCC patients samples used for IHC.

File Name: Supplementary Data 20.
Description: Scoring method based on expression of CSTB, NDRG1, PGK1, COL6A1, ITGAV, LTA4H, MB in invasive tumor front and inner OSCC.

File Name: Supplementary Data 21.
Description: Scoring on a scale of 0 to 6 to evaluate the protein abundance in invasive tumor front (ITF) and inner tumor.

File Name: Supplementary Data 22.

Description: Scoring to evaluate the protein abundance in ITF and inner tumor according to patient.

File Name: Supplementary Data 23.
Description: Association of the demographic and clinicopathological parameters with the expression of the four targeted proteins of neoplastic island from OSCC sample tissues used in IHC analysis.

File Name: Supplementary Data 24.
Description: Association of the demographic and clinicopathological parameters with the expression of three target proteins present in the stroma of OSCC sample tissues used in IHC analysis.

File Name: Supplementary Data 25.
Description: Cox multivariate analysis for local relapse of OSCC, obtained from IHC analysis.

File Name: Supplementary Data 26.
Description: Clinicopathological data from OSCC patient samples used for targeted proteomics.

File Name: Supplementary Data 27.
Description: Selection of peptides and transitions for SRM analysis.

File Name: Supplementary Data 28.
Description: The samples were divided into four sets (according to the SRM method used) and into three blocks. The samples were randomized in each block. Samples marked as "_1*" were analyzed using the first SRM method, even in the second set of samples. This is because these samples were obtained after the first set of analysis.

File Name: Supplementary Data 29.
Description: Skyline exported data for all peptides quantified in saliva cohort (12 peptides, 40 samples, technical triplicates, light & heavy peptides = 5400 rows).

File Name: Supplementary Data 30.
Description: Skyline exported data for all peptides quantified in saliva cohort (22 peptides, 40 samples, technical triplicates, light & heavy peptides = 12852 rows).

File Name: Supplementary Data 31.
Description: Patients distribution of CSTB, COL6A1, LTA4H, PGK1, ITGAV, NDRG1 abundance in SRM analysis (light-to-heavy peptide ratio= Protein abundance)

File Name: Supplementary Data 32.
Description: Association of the clinical parameters with the CSTB, LTA4H, PGK1, COL6A1, NDRG1 and ITGAV protein expression in saliva from OSCC patients.

File Name: Supplementary Data 33.
Description: Summary of datasets used for predictor generation.

File Name: Supplementary Data 34.

Description: Performance comparison of a repeated crossvalidation (100 rep. of stratified 10-fold cross-validation) on the training set (considering protein abundance) employing 7 different types of machinelearning algorithms (Linear SVM, RBF SVM, Decision Tree, Logistic Regression, Random Forest, Perceptron, and Naive Bayes).

File Name: Supplementary Data 35.
Description: Performance comparison of a repeated crossvalidation (100 rep. of stratified 10-fold cross-validation) on the training set (considering peptide abundance).employing 7 different types of machinelearning algorithms (Linear SVM, RBF SVM, Decision Tree, Logistic Regression, Random Forest, Perceptron, and Naive Bayes).

File Name: Supplementary Data 36.
Description: Summary of all possible signatures used for predictor generation using Random Forest as classifier (63 possible signatures).

File Name: Supplementary Data 37.
Description: Summary of all possible signatures used for predictor generation using Random Forest as classifier (1023 possible signatures). - Supplementary Data 38. Most relevant protein and peptide signatures considering accuracy and AUC values.

File Name: Supplementary Data 39.
Description: Comparison among potential candidates signatures.

File Name: Supplementary Data 40.
Description: Summary of all possible protein signatures used for predictor generation using Random Forest as classifier (63 possible signatures) with over-sampling.

File Name: Supplementary Data 41.
Description: Summary of all possible peptide signatures used for predictor generation using Random Forest as classifier (1024 possible signatures) with over-sampling.
