# Supplementary figures and images for: Combining discovery and targeted proteomics reveals a prognostic signature in oral cancer
Source: Nat Commun. 2018 Sep 5;9:3598. doi: 10.1038/s41467-018-05696-2 (PMC6125363; doi:10.1038/s41467-018-05696-2)

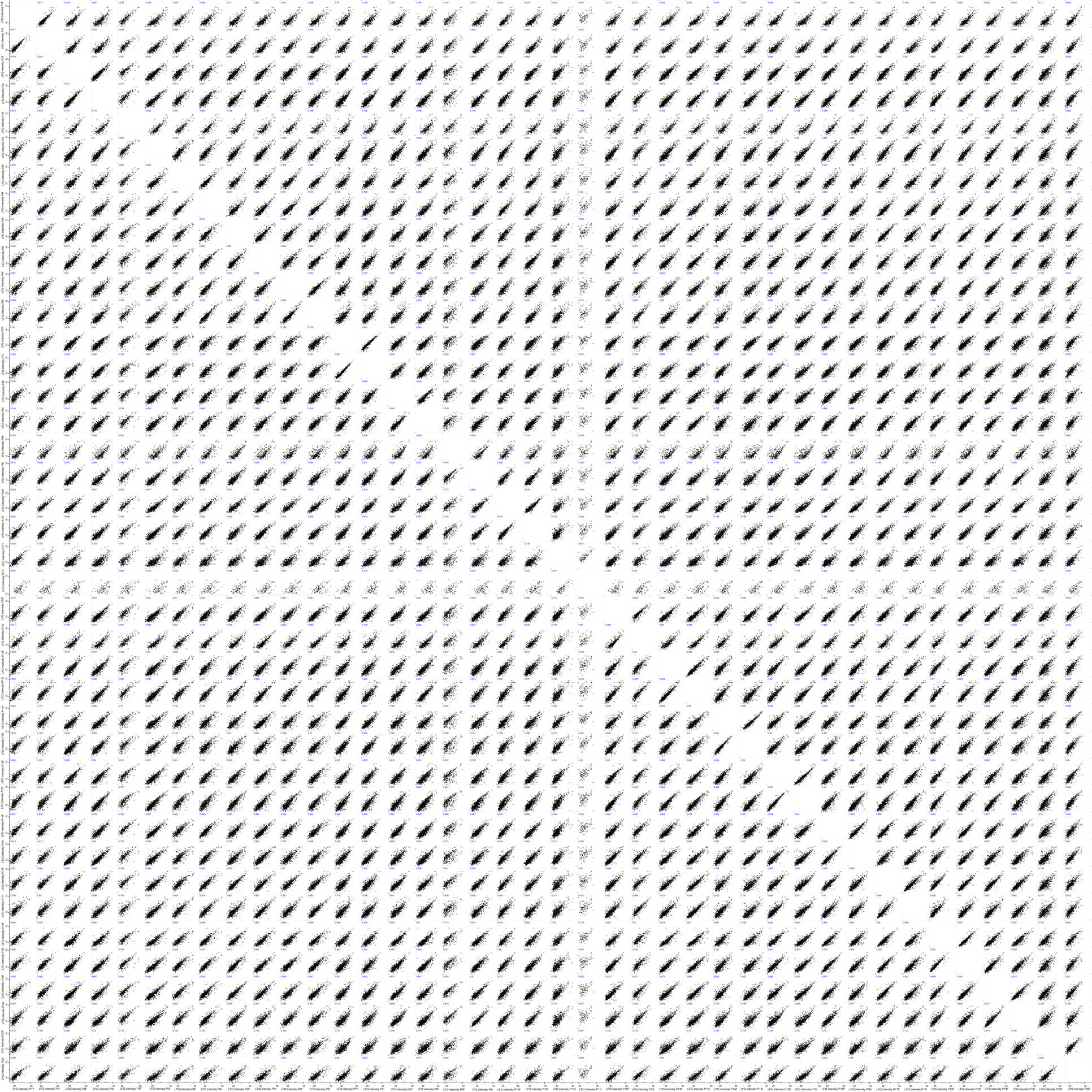

Supplement: Supplementary file 6 — Supplementary Data 3 [file 41467_2018_5696_MOESM6_ESM.pdf]

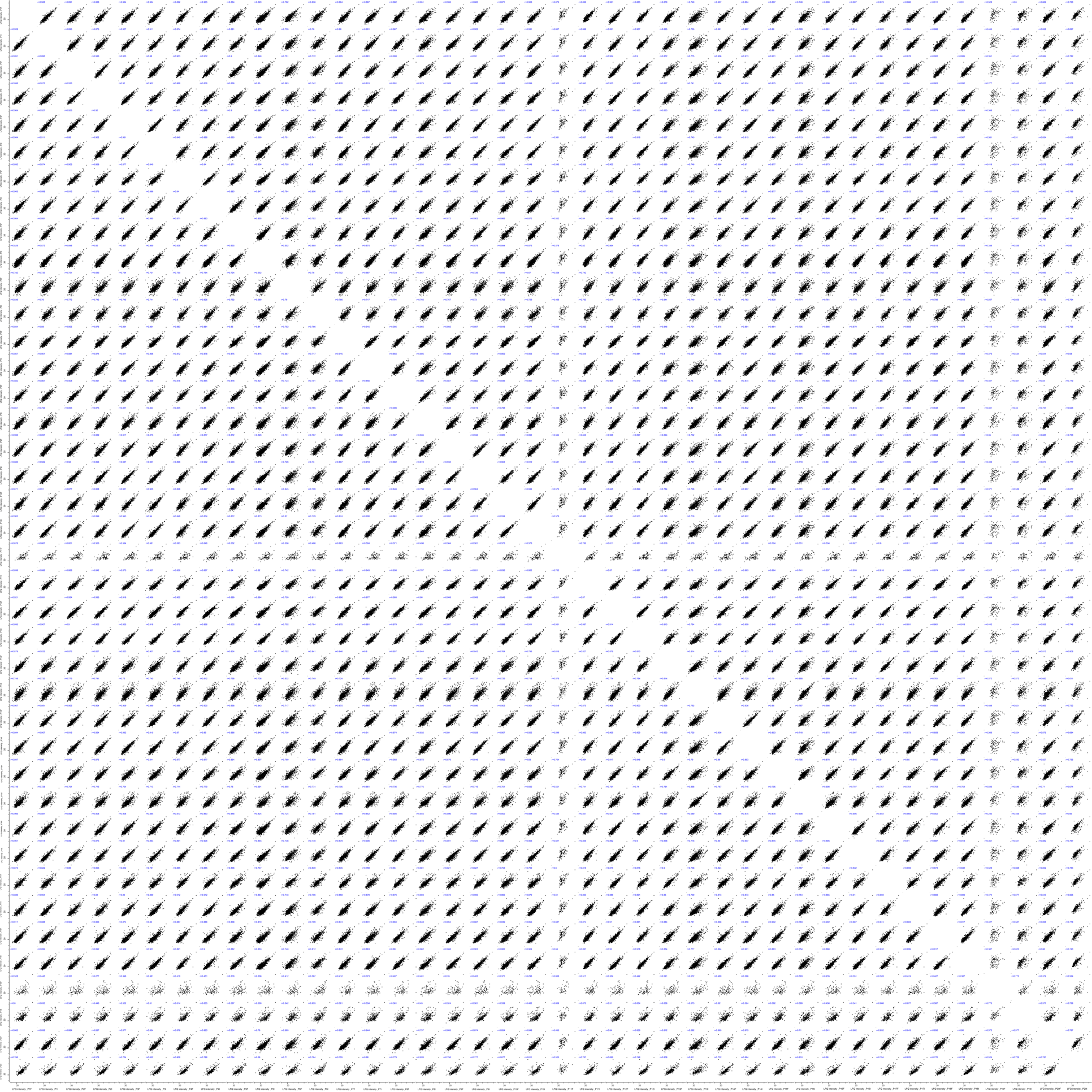

Supplement: Supplementary file 7 — Supplementary Data 4 [file 41467_2018_5696_MOESM7_ESM.pdf]
